# Enhanced uptake of potassium or glycine betaine or export of cyclic-di-AMP restores osmoresistance in a high cyclic-di-AMP *Lactococcus lactis* mutant

**DOI:** 10.1371/journal.pgen.1007574

**Published:** 2018-08-03

**Authors:** Huong Thi Pham, Nguyen Thi Hanh Nhiep, Thu Ngoc Minh Vu, TuAnh Ngoc Huynh, Yan Zhu, Anh Le Diep Huynh, Alolika Chakrabortti, Esteban Marcellin, Raquel Lo, Christopher B. Howard, Nidhi Bansal, Joshua J. Woodward, Zhao-Xun Liang, Mark S. Turner

**Affiliations:** 1 School of Agriculture and Food Sciences, University of Queensland, Brisbane, Queensland, Australia; 2 The University of Danang, University of Science and Technology, Da Nang, Vietnam; 3 Department of Microbiology, University of Washington, Seattle, WA, United States of America; 4 Monash Biomedicine Discovery Institute, Monash University, Melbourne, Australia; 5 School of Biological Sciences, Nanyang Technological University, Singapore; 6 Australian Institute for Bioengineering and Nanotechnology, University of Queensland, Brisbane, Queensland, Australia; 7 Queensland Alliance for Agriculture and Food Innovation, University of Queensland, Brisbane, Queensland, Australia; The University of Texas Health Science Center at Houston, UNITED STATES

## Abstract

The broadly conserved bacterial signalling molecule cyclic-di-adenosine monophosphate (c-di-AMP) controls osmoresistance via its regulation of potassium (K^+^) and compatible solute uptake. High levels of c-di-AMP resulting from inactivation of c-di-AMP phosphodiesterase activity leads to poor growth of bacteria under high osmotic conditions. To better understand how bacteria can adjust in response to excessive c-di-AMP levels and to identify signals that feed into the c-di-AMP network, we characterised genes identified in a screen for osmoresistant suppressor mutants of the high c-di-AMP *Lactococcus* Δ*gdpP* strain. Mutations were identified which increased the uptake of osmoprotectants, including gain-of-function mutations in a Kup family K^+^ importer (KupB) and inactivation of the glycine betaine transporter transcriptional repressor BusR. The KupB mutations increased the intracellular K^+^ level while BusR inactivation increased the glycine betaine level. In addition, BusR was found to directly bind c-di-AMP and repress expression of the glycine betaine transporter in response to elevated c-di-AMP. Interestingly, overactive KupB activity or loss of BusR triggered c-di-AMP accumulation, suggesting turgor pressure changes act as a signal for this second messenger. In another group of suppressors, overexpression of an operon encoding an EmrB family multidrug resistance protein allowed cells to lower their intracellular level of c-di-AMP through active export. Lastly evidence is provided that c-di-AMP levels in several bacteria are rapidly responsive to environmental osmolarity changes. Taken together, this work provides evidence for a model in which high c-di-AMP containing cells are dehydrated due to lower K^+^ and compatible solute levels and that this osmoregulation system is able to sense and respond to cellular water stress.

## Author summary

Second messengers relay signals received from the environment to intracellular targets that adjust cellular physiology. One widespread bacterial cyclic-dinucleotide signalling molecule, cyclic-di-AMP (c-di-AMP) has been shown to regulate a range of cellular processes via binding to protein and riboswitch targets, with most identified thus far being linked to osmoregulation functions. C-di-AMP levels need to be carefully tuned under different environmental conditions to allow optimal growth. Here we show that a *Lactococcus lactis* GdpP phosphodiesterase mutant with a high intracellular pool of c-di-AMP is able to grow under hyperosmotic conditions after acquiring mutations which increase osmolyte (potassium [K^+^] or compatible solute) uptake or by actively exporting c-di-AMP. Interestingly, elevated K^+^ or glycine betaine uptake triggered accumulation of c-di-AMP and environmental osmolarity changes were also found to significantly impact c-di-AMP levels in various bacteria. These results support a model in which c-di-AMP negatively impacts osmoresistance through inhibition of the import of osmoprotectants and this system can sense both cellular and environmental changes causing water stress.

## Introduction

In order to survive and grow, bacteria must be able to sense and respond to a multitude of environmental conditions. Changes in external osmolarity can cause cellular water loss or gain due to uncontrolled osmotic movement across the semipermeable cytoplasmic membrane. Cells can adapt to these changes in order to maintain appropriate cellular volume and solute concentration for metabolism as well as turgor pressure to drive expansion for cell division [1]. In response to an osmotic upshift (hyperosmotic stress), bacteria import potassium ions (K^+^) which is followed by a secondary response involving uptake or synthesis of compatible solutes such as glycine betaine, carnitine and proline [2]. This allows the cell to limit the loss of water and maintain turgor. During osmotic downshift (hypoosmotic stress), bacteria release K^+^ and compatible solutes from the cell in order to limit water influx causing cell swelling and in severe cases, lysis. The speed at which cells need to detect and respond to the external osmolarity change is critical and therefore the early responses in many cases involves posttranslational modulation of existing transporter activity, since the synthesis of new proteins can take too long [3]. These transporters include membrane stretch-activated mechanosensitive channels that activate during an osmotic downshift and intracellular ionic strength or K^+^ activated compatible solute uptake systems that function during an osmotic upshift [4].

The signalling molecule cyclic-di-AMP (c-di-AMP) found in many Gram-positive bacteria and some Gram-negative bacteria has been recently demonstrated to play a significant role in regulating K^+^ and compatible solute (carnitine) import systems either via direct binding to transporter protein complexes (Ktr and OpuCA), a regulatory sensor kinase (KdpD) or riboswitch upstream of the transporter genes (*ktr* and *kimA*) [5–9]. A high level of c-di-AMP has been found to repress K^+^ and carnitine uptake, thereby inhibiting growth under hyperosmotic conditions [5, 6, 10, 11]. Conversely, low/absent c-di-AMP results in cells which have likely uncontrolled K^+^ and compatible solute uptake, resulting in viability only under conditions where external K^+^ and compatible solute concentrations are low or when the cells are osmotically stabilised by the addition of high salt [9, 12].

Whilst the role of c-di-AMP in osmoregulation is becoming evident, the signals which trigger changes in the c-di-AMP pool are still poorly understood [13, 14]. C-di-AMP is synthesised from two ATP molecules by diadenylate cyclase (DAC) enzymes and degraded by phosphodiesterase (PDE) enzymes. In most Gram-positive bacteria, only one DAC exists, named CdaA or DacA, and it is localised to the membrane via three transmembrane domains [15]. Most bacteria also contain one PDE, called GdpP, while others contain an additional PDE called PgpH [16] and both of these PDEs are membrane localised [17]. It is through these enzymes, and possibly active c-di-AMP export, that the c-di-AMP pool is regulated. The intracellular level of c-di-AMP is under strict control since both low and high levels have been shown to be detrimental to growth in several Firmicutes [18, 19]. C-di-AMP levels in bacteria have been found to be responsive to growth phase, acid stress, growth media nitrogen source, (p)ppGpp induced by mupirocin, mutations in the peptidoglycan biosynthesis enzyme GlmM or YbbR protein and inactivation of the LiaFSR membrane stress response system [11, 20–23]. Upregulated CdaA expression and a 2-fold higher level of c-di-AMP was observed in *Bacillus subtilis* grown in defined media with 5mM K^+^ compared to cells grown with 0.1mM K^+^ [9]. Recently, it was found that inactivation of the gating component (CabP) of the Trk family K^+^ transporter in *Streptococcus pneumoniae* reduced the c-di-AMP level [24]. These results suggest that K^+^ may act as a signal for c-di-AMP level modulation directly or indirectly.

In this study, we sought to better understand the cause of high c-di-AMP toxicity and to identify mechanisms by which this toxicity can be averted in bacteria. We used a high c-di-AMP containing *Lactococcus lactis* Δ*gdpP* mutant which is hypersensitive to elevated salt in growth media. This strain can accrue suppressor mutations which allows it to grow under such an osmotically stressful condition and previous work has identified changes in the c-di-AMP synthase CdaA and binding partner GlmM which both lowered the c-di-AMP level [11]. Here we expanded the screen to saturation and identified and characterised three different suppressor mutant groups. Two groups rescue osmoresistance through mutations which elevate osmolyte (K^+^ or glycine betaine) uptake while one group activated the expression of a c-di-AMP export system involving a multidrug resistance protein (MDR) which lowered intracellular c-di-AMP. In addition we show that c-di-AMP accumulates in response to elevated K^+^ and glycine betaine uptake, and in several bacteria the c-di-AMP level is rapidly responsive to environmental osmolarity changes, thus allowing it to sense and respond to water stress.

## Results

### Suppressor mutations restore osmoresistance in Δ*gdpP* by c-di-AMP level dependent and independent means

To identify regulators of the c-di-AMP pool, we previously employed a genetic screen to obtain osmoresistant suppressors of the high c-di-AMP *Lc*. *lactis* Δ*gdpP* strain OS2 [11]. In the current study, a further 212 osmoresistant suppressor mutants were obtained and analysed. Numerous *cdaA* mutations were identified (n = 184) as well as several restorative *gdpP* mutations (n = 8). Twenty suppressors which contained identical *cdaA* and *gdpP* sequences as the parent strain were subjected to whole genome sequencing and the mutation locations are shown in Table 1. The 20 suppressors contained mutations in 3 functionally linked gene groups. The first group included 4 independent mutants which possessed changes in the K^+^ uptake transporter KupB (Llmg0588). The second group included 11 independent mutants which contained changes in the Eep/PptAB hydrophobic peptide processing and export system (S1A Fig). Eep (Llmg2413) is a transmembrane metalloprotease homolog of RseP [25] and PptAB (Llmg2271-2270) are ATPase and permease homologs of EcsAB [26]. Interestingly one suppressor contained a mutation in *pptB* and a mutation in *busR* which encodes a transcriptional repressor of the glycine betaine transporter [27]. The third group included 2 independent mutants which contain deletions in an intergenic region between the ribosomal 50S protein encoding *rplL* and the MarR family transcriptional regulator encoding *rmaX*.

**Table 1:** Mutations identified in osmoresistant suppressor mutants of Δ*gdpP*.

Suppressor number	KupB K^+^ importer (Llmg0588)	Eep metalloprotease RseP (Llmg2413)	PptAB peptide ABC transporter EscAB (Llmg2271-Llmg2270)	BusR Glycine betaine transporter transcriptional repressor (Llmg1047)	Other mutations
1	A618V				
2	R508G				
3	F331V				FtsX cell division protein (P249L) (Llmg1545*)*
4	F635L		*pptA* S55fs^1^		
5, 6, 7		G188C			
8		G188C			phosphotransferase (S93fs) (Llmg1812)
9		G31fs			
10		Y56*^2^			
11		N215fs			
12		L195fs			A deletion in intergenic sequence 36bp downstream of *llmg1029*
13			*pptA* Q139P		
14			*pptB* I227fs		
15			*pptB* S189fs	126-bp in-frame deletion (removes 42 aa including G42 to Q83)	
16			*pptB* A109fs		Mevalonate kinase (Llmg0425) H21D
17			19kb deletion (from *llmg2251* to *llmg2272* encompassing *pptAB*)		
18, 19					209bp intergenic deletion downstream of *rplL* (*llmg1208*)
20					85bp intergenic deletion downstream of *rplL* (*llmg1208*)

^1^ fs = frameshift

^2^ = stop codon

**Fig 1:**
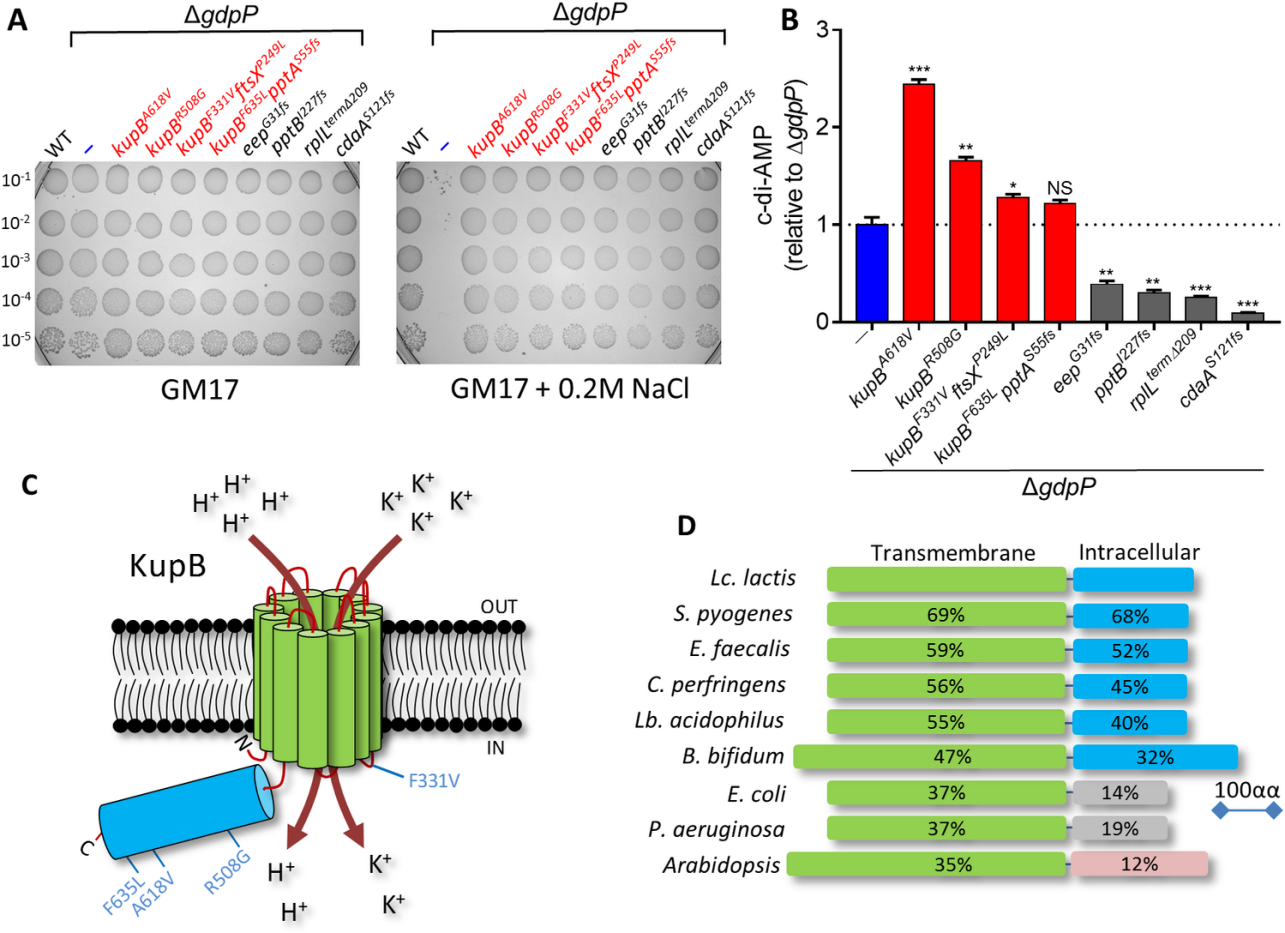
Mutations in *kupB* restore osmoresistance to *Lc*. *lactis* Δ*gdpP* independently of c-di-AMP. **(A)** Comparison of growth of *Lc*. *lactis* MG1363 background strains on GM17 agar or GM17 agar + 0.2M NaCl following spotting of serial dilutions. **(B)** Levels of c-di-AMP (mean ± SEM) in *Lc*. *lactis* strains from three independent biological replications. *P* < 0.001 (***), *P* < 0.01 (**) and *P* < 0.05 (*) indicate significant differences compared to Δ*gdpP* (one-way ANOVA followed by Student’s t test). NS = not significant. **(C)** Location of suppressor mutations in KupB. **(D)** Amino acid identities of the two Kup domains in other organisms compared to *Lc*. *lactis* KupB.

Osmoresistance of representative strains from these groups as well as a *cdaA* mutant are shown in Fig 1A. As found in previous work [11], mutations lowering the c-di-AMP level restored osmoresistance in Δ*gdpP* and this was found to be the case for the *eep*, *pptB*, *rpilL*^*Δterm209*^ and *cdaA* mutants (Fig 1B). A *pptB* gene disruption was constructed in the Δ*gdpP* background strain which, as expected, resulted in restoration of osmoresistance (S1B Fig) and a lowering of c-di-AMP (S1C Fig). Strikingly, the *kupB* mutants were found to have significantly elevated c-di-AMP or at least equivalent levels to the Δ*gdpP* parent (Fig 1B). This is the only group of osmoresistant suppressors we have identified thus far which do not have lower c-di-AMP levels relative to the Δ*gdpP* parent. Therefore, they have developed osmoresistance independently of c-di-AMP pool modulation. Importantly suppressors which contain only a single mutation in *kupB* (*kupB*^*A618V*^ and *kupB*^*R508G*^) had significantly elevated c-di-AMP (Fig 1B), suggesting that changes in K^+^ uptake triggers c-di-AMP accumulation. In the two other *kupB* suppressors, one additional mutation is present in each (*ftsX*^*P249L*^ or *pptA*^*S55fs*^) which likely caused a lowering of the c-di-AMP level (Fig 1B).

### Elevated K^+^ uptake by KupB rescues osmoresistance of Δ*gdpP* and triggers accumulation of c-di-AMP

KupB contains twelve transmembrane spanning domains and a C-terminal 227 amino acid intracellular domain (Fig 1C). Among the 4 identified KupB missense mutations in the suppressor mutants, one is present in an internal loop region between membrane-spanning domains while three are in the C-terminal intracellular domain. KupB is homologous to a large family of Kup proteins present in several Gram-positive bacteria, Gram-negative bacteria and plants (Fig 1D) where their roles in K^+^ uptake and osmoregulation is well established [28]. Kup proteins possess highly similar transmembrane domains, but differ in their C-terminal intracellular domain (Fig 1D) which has been proposed to regulate K^+^ uptake [29]. Mutations identified in our suppressor screen restore osmoresistance, suggesting that they are gain of function mutations and might result in increased K^+^ uptake most likely through modification of channel regulation.

**Fig 2:**
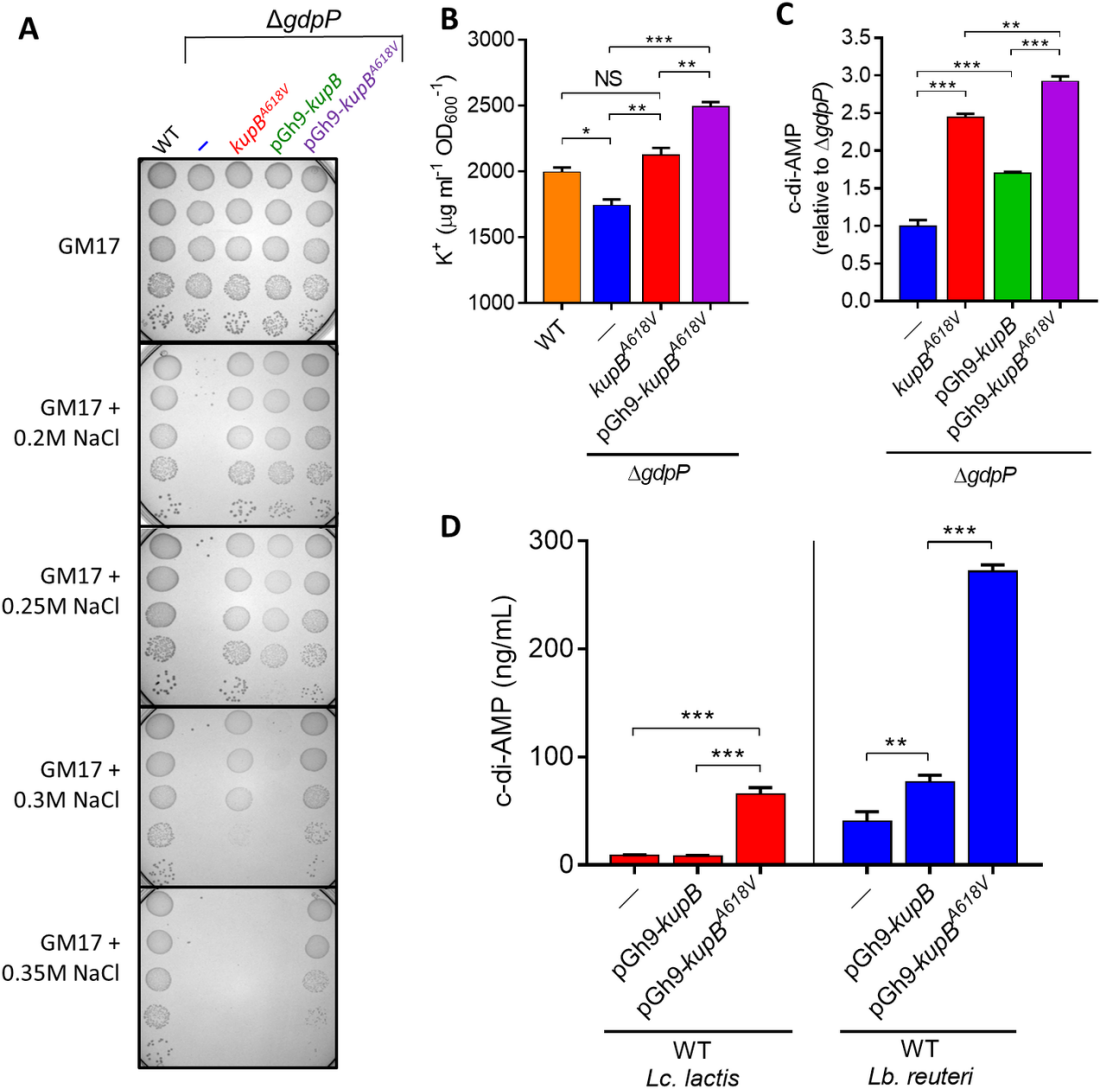
Constitutive KupB activity results in higher salt resistance and c-di-AMP levels in bacteria. **(A)** Overexpression of *kupB* and *kupB*^*A618V*^ in *Lc*. *lactis* Δ*gdpP* enhances osmoresistance. Spots (5μl) are from log-phase cells diluted from 10^−1^ (top) to 10^−5^ (bottom). **(B)** K^+^ levels in WT *Lc*. *lactis*, Δ*gdpP*, Δ*gdpPkupB*^*A618V*^ and Δ*gdpP* overexpressing *kupB*^*A618V*^. **(C)** C-di-AMP levels in Δ*gdpP* and Δ*gdpPkupB*^*A618V*^ and Δ*gdpP* overexpressing *kupB* and *kupB*^*A618*^. **(D)** C-di-AMP levels in WT *Lc*. *lactis* and WT *Lb*. *reuteri* overexpressing *kupB* and *kupB*^*A618V*^. Data are the mean ± SEM from three independent biological replications. Significant results *P* < 0.001 (***), *P* < 0.01 (**) and *P* < 0.05 (*) are indicated (one-way ANOVA followed by Student’s t test).

To determine if the KupB mutations resulted in a gain-of-function activity we overexpressed KupB^A618V^ and wild-type KupB using its native promoter in Δ*gdpP* and measured their effect on osmoresistance. It was found that overexpression of wild-type KupB increased the osmoresistance of Δ*gdpP*, suggesting that an increase in *kupB* copy number can increase K^+^ uptake (Fig 2A). Overexpression of KupB^A618V^ in Δ*gdpP* restored osmoresistance to a higher level than both Δ*gdpP* overexpressing wild-type KupB and the Δ*gdpPkupB*^*A618V*^ suppressor mutant (Fig 2A). We next determined the intracellular K^+^ levels and found that Δ*gdpP* has lower K^+^ compared to both the wild-type and the Δ*gdpPkupB*^*A618V*^ suppressor mutant (Fig 2B). Overexpression of KupB^A618V^ in Δ*gdpP* resulted in 43% higher K^+^ level than Δ*gdpP* (Fig 2B). Together these results confirm that the A618V mutation in KupB is a gain-of-function mutation which increases osmoresistance by increasing K^+^ uptake.

One possibility was that these mutations prevent c-di-AMP from binding KupB and inhibiting K^+^ uptake, since it has been shown to bind to and/or regulate K^+^ transporters in other bacteria such as Ktr, Kdp and KimA. To determine if c-di-AMP binds to KupB we carried out the differential radial capillary action of ligand assay (DRaCALA) using the KupB intracellular C-terminal as fusions to hexa-His or MBP tags. This domain could be expressed but both fusion proteins were insoluble and testing of whole cell lysates with radiolabelled c-di-AMP did not reveal any binding (S2 Fig). Previous work has shown that insoluble proteins in whole cell lysates can bind cyclic dinucleotides [8, 30]. Therefore at this stage the regulation of KupB by c-di-AMP or other signals is not known. Interestingly using quantitative reverse transcriptase PCR (qRT-PCR), it was found that transcription of *kupB* was slightly higher (3-fold; *P* = 0.02; Student’s t test) in Δ*gdpP* compared to wild-type. This may be in response to low intracellular K^+^ levels present in the high c-di-AMP mutant Δ*gdpP*.

The c-di-AMP levels in *L*. *lactis* Δ*gdpP* strains overexpressing wild-type KupB or KupB^A618V^ were determined next. Overexpression of wild-type KupB triggered higher c-di-AMP in Δ*gdpP*, while the c-di-AMP level in Δ*gdpP* overexpressing KupB^A618V^ was even higher (Fig 2C). The level of c-di-AMP in these Δ*gdpP* background strains directly correlated with their osmoresistance level (Fig 2A), with the highest c-di-AMP strains being the most osmoresistant. This result is in stark contrast to previous work, where high c-di-AMP Δ*gdpP* mutants were found to be osmosensitive due to reduced activity or expression of K^+^ and/or compatible solute transporters [5, 7, 8, 11]. In the situation here, it appears that increased intracellular K^+^ due to enhanced K^+^ import activity not only results in the restoration of osmoresistance in Δ*gdpP*, but also that intracellular K^+^ serves either directly or indirectly as a signal for the cell to increase the c-di-AMP level. Indeed the level of K^+^ in Δ*gdpP*, Δ*gdpPkupB*^*A618V*^ and Δ*gdpP*-pGh-*kupB*^*A618V*^ strains directly correlated with the level of c-di-AMP (Fig 2B and 2C). An increase in the c-di-AMP level can occur via either increased c-di-AMP synthesis and/or decreased c-di-AMP hydrolysis. However it appears that control is mediated by increased c-di-AMP synthesis by CdaA here, as *gdpP* is defective in these strains. Using qRT-PCR, transcription of *cdaA* in Δ*gdpPkupB*^*A618V*^ was unchanged compared with the parent Δ*gdpP* (*P* = 0.96; Student’s t test), which suggests that increased c-di-AMP levels are not due to higher *cdaA* expression.

Next we examined if elevated K^+^ uptake could trigger greater c-di-AMP accumulation in strains other than Δ*gdpP*. We overexpressed KupB and KupB^A618V^ in wild-type *Lc*. *lactis* and related *Lactobacillus reuteri* which both contain GdpP. In *Lc*. *lactis*, the level of c-di-AMP was 7.5-fold higher when KupB^A618V^ was overexpressed compared to cells overexpressing KupB or the wild-type (Fig 2D). In wild-type *Lb*. *reuteri*, overexpression of KupB and KupB^A618V^ resulted in 1.9-fold and 6.7 fold c-di-AMP level increases, respectively (Fig 2D). Therefore the c-di-AMP pool size correlates directly with K^+^ uptake activity in at least two different bacterial genera irrespective of the presence of GdpP.

### BusR regulates glycine betaine uptake in response to the c-di-AMP level and excess glycine betaine triggers c-di-AMP accumulation

**Fig 3:**
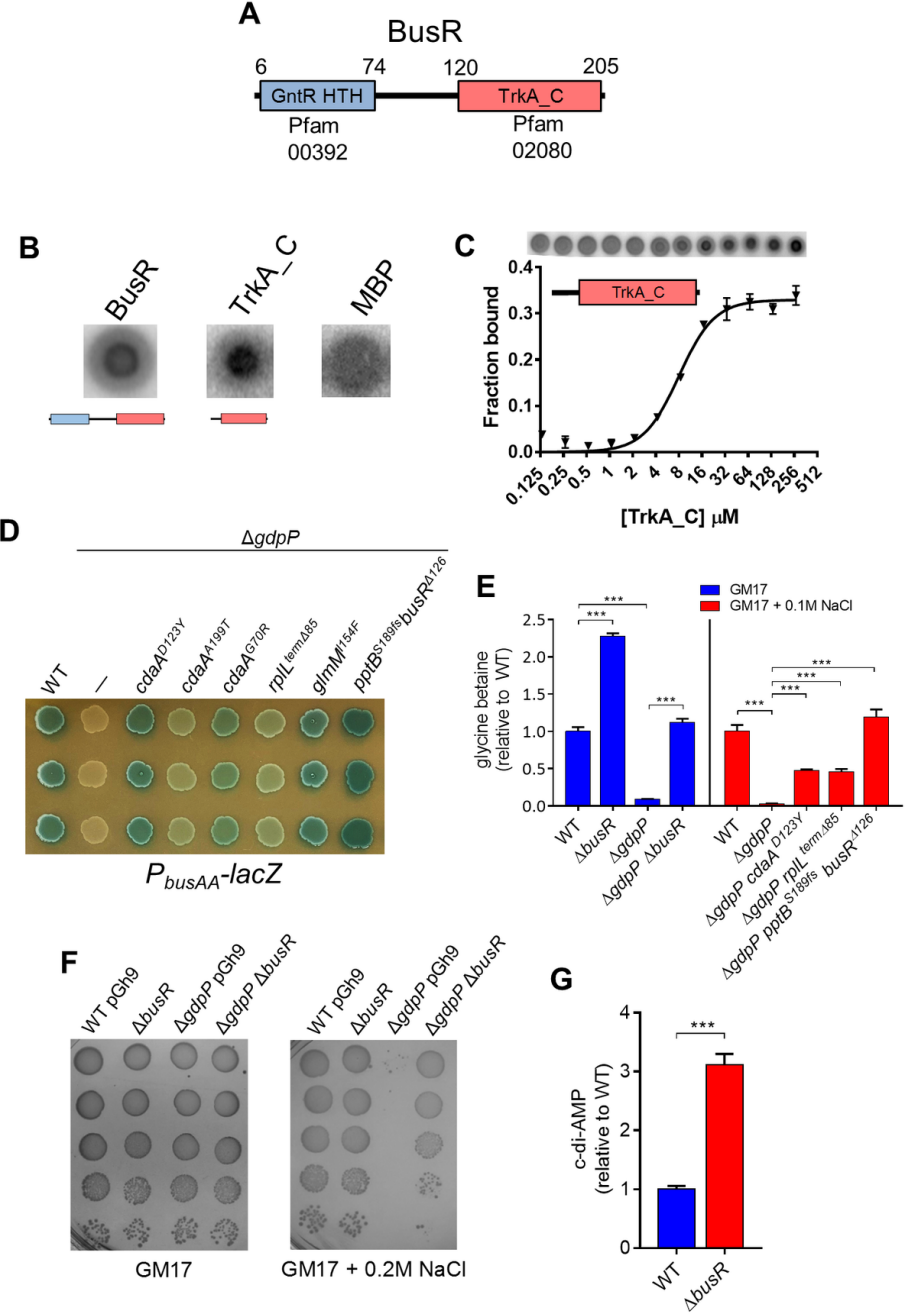
BusR binds c-di-AMP and exacerbates the osmosensitive phenotype of Δ*gdpP*. **(A)** Domains in BusR. **(B)** DRaCALA of cell lysates containing full length BusR, the BusR TrkA_C domain or MBP with ^32^P-c-di-AMP. **(C)** DRaCALA and binding affinity of the purified BusR TrkA_C domain with ^32^P-c-di-AMP. **(D)** Expression of *busAA* promoter as a *lacZ* fusion in different *Lc*. *lactis* strains on GM17 agar + 0.1M NaCl. **(E)** Intracellular glycine betaine levels. Strains in red bars were grown in GM17 + 0.1M NaCl due to the osmoresistant suppressor Δ*gdpPcdaA*^*D123Y*^ growing poorly in GM17 alone. Data are the mean ± SEM from three independent biological replications. *P* < 0.001 (***) indicate significant differences compared to Δ*gdpP* (one-way ANOVA followed by Tukey’s test). **(F)** Comparison of growth of strains on GM17 agar or GM17 agar + 0.2M NaCl following spotting of serial dilutions. **(G)** C-di-AMP levels in WT *Lc*. *lactis* and Δ*busR*. Data are the mean ± SEM from three independent biological replications. Significant results *P* < 0.001 (***) are indicated (Student’s t test).

One suppressor mutant contained an in-frame deletion in the *busR* gene and an inactivating mutation in the *pptB* gene (S189fs) (Table 1). This *busR* deletion removed 42 residues (amino acids 42–83) from the N-terminal GntR HTH domain (Fig 3A). BusR is a transcriptional repressor of the BusAA-AB glycine betaine transporter in *Lc*. *lactis* [27]. To determine if the 42 amino acid deletion reduces the ability of BusR to repress the *busAA* promoter, we expressed the wild-type and mutant BusR in *E*. *coli* in conjunction with a *lacZ* fusion to the promoter of *busAA*. It was found that the *busAA* promoter activity increased moderately upon NaCl addition, similarly to that described before [31] (S3A Fig). The wild-type BusR repressed expression of *busAA* more strongly than the mutated BusR under all conditions with or without additional NaCl (S3A Fig) suggesting that the 42 amino acid deletion is a loss-of-function mutation. BusR is a member of the family of GntR regulators and homologs are present in a subset of Firmicutes including *Clostridium* spp. and certain lactic acid bacteria (S3B Fig). These proteins contain a C-terminal TrkA_C domain (Pfam02080) which is also found in gating components of the Trk family of K^*+*^ transporters that bind c-di-AMP [6, 7]. To determine if BusR binds c-di-AMP we expressed both full length and the TrkA_C domain in *E*. *coli* and carried out DRaCALA on whole cell lysates. Both full length BusR and the BusR TrkA_C domain bound c-di-AMP (Fig 3B) and the purified TrkA_C domain was found to interact with c-di-AMP with a K_d_ of ~10μM (Fig 3C). We determined the expression of *busAA-AB* in strains with varying c-di-AMP levels using a plasmid containing the *busAA-AB* promoter fused to a *lacZ* reporter. It was found that expression of *busAA-AB* was absent in the high c-di-AMP strain Δ*gdpP* compared to wild-type (Fig 3D). All osmoresistant suppressors were found to have greater *busAA-AB* expression as compared to Δ*gdpP* (Fig 3D). Suppressors which have lower c-di-AMP including those with mutations in *cdaA*, *rpilL*^*Δterm85*^ and *glmM* had restored *busAA-AB* expression to varying levels (Fig 3D). The suppressor mutant Δ*gdpP pptB*^*189fs*^ *busR*^*Δ126*^ containing an inactive BusR showed very high *busAA-AB* expression, as expected (Fig 3D). Glycine betaine levels in different strains were measured and it was found that Δ*gdpP* contained ~10-fold less than wild-type (Fig 3E). Inactivation of *busR* in the wild-type or Δ*gdpP* background resulted in elevated glycine betaine levels compared to their parent strains (Fig 3E). Glycine betaine levels in osmoresistant suppressor mutants of Δ*gdpP* (*cdaA*^*D123Y*^, *rpilL*^*Δterm85*^, *pptB*^*189fs*^*busR*^*Δ126*^) were all significantly higher (14- to 37-fold) compared with Δ*gdpP* (Fig 3E). These results suggest that BusAA-AB expression and glycine betaine levels are reduced in strains with high c-di-AMP due to this signalling molecule binding to BusR and enhancing its repression.

Next we determined if strong repression of glycine betaine transporter expression by BusR contributes to the osmosensitive phenotype of Δ*gdpP*. Inactivation of BusR in Δ*gdpP* restored osmoresistance, indicating that glycine betaine transport allows the Δ*gdpP* to regain normal turgor pressure under high osmolarity conditions (Fig 3F). Lastly we investigated if increased glycine betaine uptake caused by loss of BusR triggers c-di-AMP accumulation. It was found that the level of c-di-AMP was indeed 3-fold higher in Δ*busR* compared to WT (Fig 3G). Taken together these results provide evidence that uncontrolled glycine betaine uptake is sensed by the cell, which in turn responds by elevating the c-di-AMP level in order to enhance BusR mediated repression of *busAA-AB*.

### Mutations activating the expression of a MDR restores osmoresistance of Δ*gdpP* by lowering the intracellular c-di-AMP level through active export

**Fig 4:**
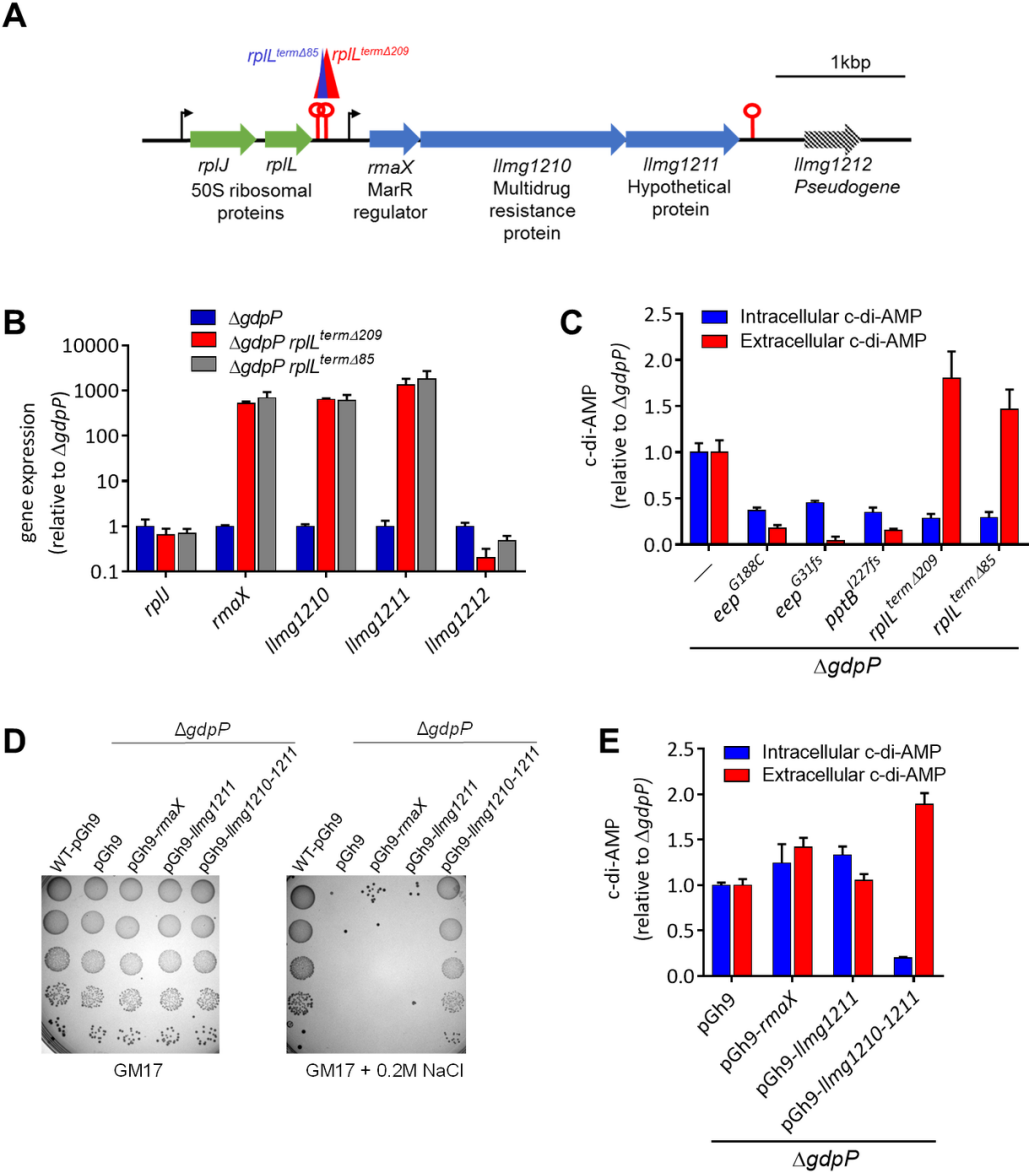
Active export of c-di-AMP lowers intracellular c-di-AMP and restores osmoresistance in *Lc*. *lactis* Δ*gdpP*. **(A)** Location of two independent intergenic deletions between *rplL* and *rmaX*. Arrows and lollipops indicate predicted transcription start sites and terminators, respectively. **(B)** Expression of genes in Δ*gdpP* and the two suppressor mutants containing intergenic deletions (Δ*gdpP rplL*^*term*Δ209^ and Δ*gdpP rplL*^*term*Δ85^) using qRT-PCR. **(C)** Levels of intracellular and extracellular c-di-AMP in Δ*gdpP* and osmoresistant suppressor derivatives. Data are the mean ± SEM from three independent biological replications. **(D)** Comparison of growth of strains on GM17 agar or GM17 agar + 0.2M NaCl following spotting of serial dilutions. Includes Δ*gdpP* strains overexpressing different genes in pGh9 plasmid using the promoter from *rplJ*. **(E)** Levels of intracellular and extracellular c-di-AMP in Δ*gdpP* strains overexpressing different genes from the pGh9 plasmid using the promoter from *rplJ*. Data are the mean ± SEM from three independent biological replications.

Two independent osmoresistant mutants containing overlapping 209 and 85bp deletions in an intergenic region between *rplL* and *rmaX* were obtained (Figs 4A and S4A). This deleted region includes two inverted repeats with free energies of -8.1 and -11.7 kcal/mol (S4A and S4B Fig). Either both or one are likely to act as transcription terminators for the upstream *rplJ*-*rplL* operon. The two different deletion mutants showed equivalent salt resistance and the deletion events were verified by PCR using primers flanking the deletion (S4C and S4D Fig). The operon upstream of the deletion events contains two ribosomal genes *rplJ* (50S ribosomal protein L10) and *rplL* (50S ribosomal protein L7/L12) (Fig 4A). Downstream of the deletion is a three gene operon composed of *rmaX* (MarR transcriptional regulator), *llmg1210* (MDR of the EmrB family) and *llmg1211* (predicted membrane protein of unknown function). Due to the deletion of a putative terminator, we hypothesised that transcription of the downstream operon may now be increased due to extension of transcripts from the likely highly expressed ribosomal genes. Using qRT-PCR, it was found that RNA transcripts of *rmaX*, *llmg1210* and *llmg1211* were several hundred fold higher in the two intergenic deletion suppressor mutants relative to the parent Δ*gdpP* (Fig 4B). As expected, expression of the *rplJ* and *llmg1212* were not elevated in the deletion suppressor mutants (Fig 4B).

Previous work has demonstrated that c-di-AMP is actively secreted by MDR proteins of the MFS superfamily in *L*. *monocytogenes* and *B*. *subtilis* [17, 32–36]. Llmg1210 was found to share 45% amino acid identity with MdrT (Lmo2588) and 36% identity with MdrM (Lmo1617) (S5 Fig), both of which have been shown to export c-di-AMP in *L*. *monocytogenes* [32]. We hypothesised that higher MDR expression leads to greater c-di-AMP export resulting in a lowering of the intracellular c-di-AMP. The 2 different deletion suppressor mutants contained reduced intracellular c-di-AMP levels compared to Δ*gdpP*, which were equivalent to that of *eep* and *pptB* suppressor mutants (Fig 4C). However extracellular c-di-AMP levels were found to be disproportionately high in the deletion mutants, between 7 and 45 fold higher compared to the *eep* and *pptB* suppressor mutants (Fig 4C). To determine which gene(s) within the overexpressed operon is required for c-di-AMP export and osmoresistance, we overexpressed each using the predicted strong *rplJ* promoter on a plasmid in the Δ*gdpP* strain. It was found that overexpression of *rmaX* or *llmg1211* did not lower the intracellular c-di-AMP level or restore osmoresistance (Fig 4D and 4E). We were able to clone *llmg1210* downstream of the *rplJ* promoter in *E*. *coli* as a host, however upon introduction in *Lc*. *lactis* Δ*gdpP*, mutations in *llmg1210* occurred in several independent trials, suggesting that overexpression of this protein by itself is toxic. When we cloned *llmg1210* combined with *llmg1211* downstream of the *rplJ* promoter, the plasmid was stable in Δ*gdpP* and a reduction of intracellular c-di-AMP occurred along with rescue of osmoresistance (Fig 4D and 4E).

To further confirm the role of this overexpressed operon in c-di-AMP export, we generated a series of chromosomally integrated mutants throughout the operon in the osmoresistant intergenic deletion suppressor strain Δ*gdpPrplL*^*termΔ85*^ (S6A Fig). In this strain, the *rmaX-llmg1210-llmg1211* operon is highly expressed. It was found that inactivation of expression of the entire operon by plasmid insertion elevated intracellular c-di-AMP and eliminated osmoresistance (S6B and S6C Fig). Interestingly the c-di-AMP level was significantly higher than that in Δ*gdpP* suggesting that the *rmaX* operon may be expressed and function to export c-di-AMP in Δ*gdpP* without the deletion event. Insertion of a plasmid allowing overexpression of *rmaX* only also resulted in similarly high c-di-AMP level and osmosensitivity. Plasmid insertion allowing overexpression of *rmaX*-*llmg1210* from the genome lowered the c-di-AMP level compared to the two strains with plasmid insertions upstream (*p* < 0.001), however this was not sufficiently low enough to restore osmoresistance (S6B and S6C Fig). Only the plasmid insertion following *llmg1211*, which allows expression of all three genes, lowered the intracellular c-di-AMP level enough to restore osmoresistance in this deletion suppressor mutant (S6B and S6C Fig). Together these results suggest that the MDR *llmg1210* is a c-di-AMP export protein, but requires *llmg1211* for full activity and/or stability.

### Environmental osmolarity changes trigger rapid c-di-AMP pool adjustment

**Fig 5:**
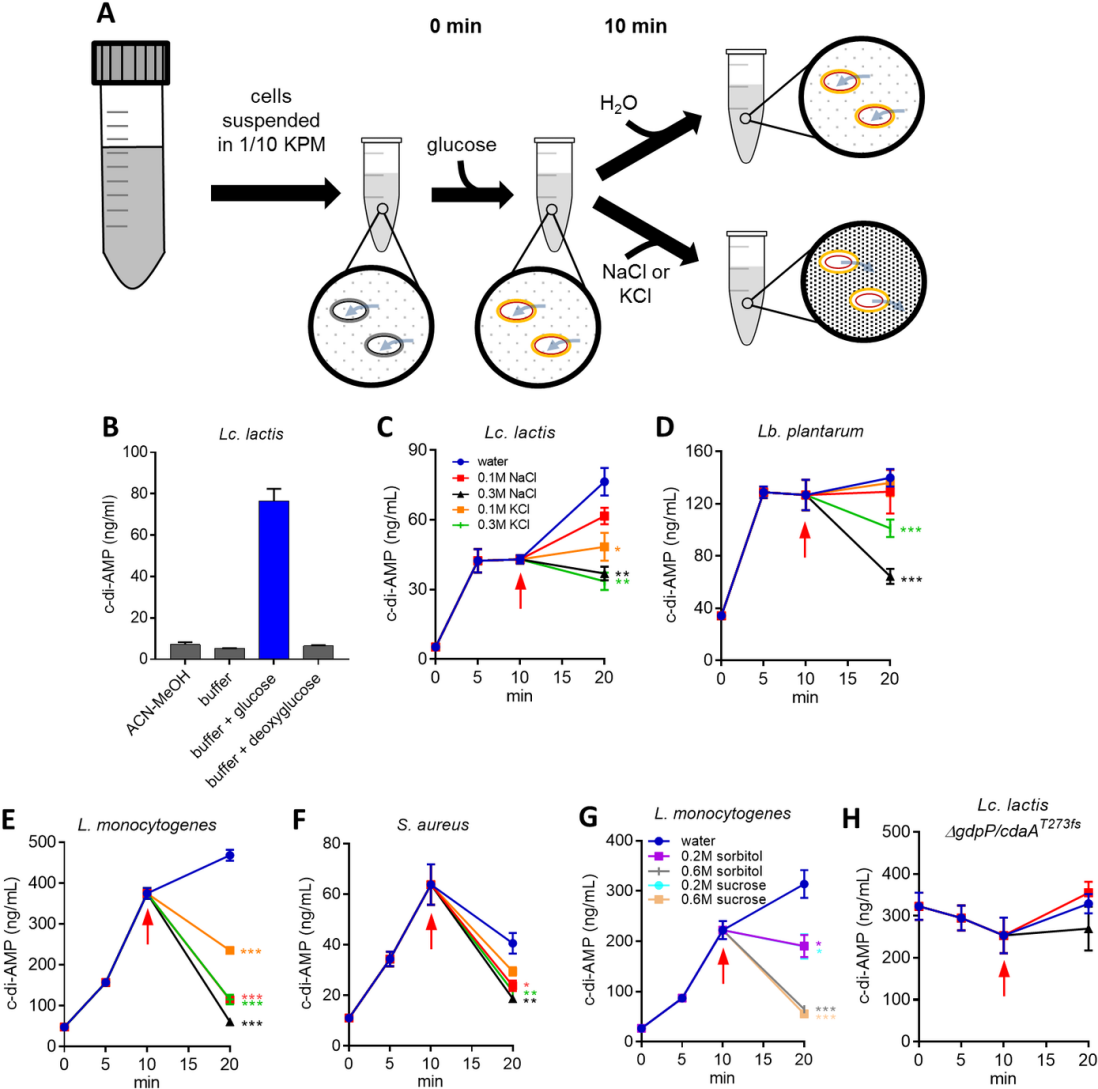
C-di-AMP levels in non-growing energised cells respond rapidly to environmental osmolarity changes. **(A)** Schematic diagram showing the steps in the non-growing cell suspension assay. Cells are suspended in a low osmolarity buffer causing water influx. Glucose is added to energise the cells at time 0 to provide ATP for c-di-AMP synthesis. After 10 mins, either water or NaCl/KCl is added causing further cellular hydration or cellular dehydration, respectively. **(B)** C-di-AMP levels in *Lc*. *lactis* WT suspended in different solvents/solutions: ACN-MeOH (acetonitrile:methanol) or buffer (1/10 KPM) with and without glucose and deoxyglucose. **(C)** C-di-AMP levels in *Lc*. *lactis* WT cells in 1/10 KPM buffer + glucose. After the 10 min (arrow), cells were treated with water, 0.1M NaCl, 0.3M NaCl, 0.1 KCl and 0.3M KCl and c-di-AMP levels were measured at 20 mins. The same experiment was carried out for cells of *Lb*. *plantarum* **(D)**, *L*. *monocytogenes* **(E)**, *S*. *aureus* **(F)**, and *Lc*. *lactis* Δ*gdpP/cdaA*^*T273fs*^ **(H)** with the key for treatments the same as that shown in **(C)**. Non-ionic treatments (sucrose and sorbitol) were also performed using *L*. *monocytogenes* **(G)**. Mean ± SEM levels of c-di-AMP were measured from three independent cell suspensions. *P* < 0.001 (***), *P* < 0.01 (**) and *P* < 0.05 (*) indicate significant differences at the 20 minute time point relative to the water addition sample (one-way ANOVA followed by Student’s t test).

From the findings above and from other work, it is clear that c-di-AMP is a major regulator of osmoresistance. Therefore the c-di-AMP pool size would be predicted to be responsive to and inversely proportional to the external osmolarity allowing appropriate regulation of K^+^ and compatible solute transporters to control cell turgor. Therefore cells experiencing high turgor in low osmolarity environments would elevate their c-di-AMP pool, while cells experiencing low turgor in high osmolarity environments would deplete their c-di-AMP pool. Difficulties exist when trying to test this hypothesis however, since wild-type bacteria generally have low levels of c-di-AMP and changes in environmental osmolarity imposed during growth will likely lead to other physiological and gene expression changes which may have indirect influences on the c-di-AMP pool size. In order to separate direct and indirect effects, a simple new method was developed whereby the c-di-AMP level could be monitored in non-growing cells in low and high osmolarity conditions (Fig 5A). In an initial attempt to stimulate c-di-AMP accumulation, we suspended washed *Lc*. *lactis* WT cells in a low osmolarity buffer to stimulate increased turgor pressure. However, the c-di-AMP level remained the same as that found in cells grown in culture media and extracted with acetonitrile-methanol (Fig 5B). We hypothesised that washed cells have depleted ATP and therefore no immediate precursor for c-di-AMP synthesis. This is the case for bacterial ATP binding cassette (ABC) solute uptake systems which need to be energised by glucose addition to cells during uptake assays [37, 38]. It was found that energizing the cells by the addition of glucose resulted in a ~10-fold increase in c-di-AMP (Fig 5B). The addition of the closely related analog deoxyglucose, which is unable to initiate glycolysis, did not trigger c-di-AMP accumulation (Fig 5B). Several Gram-positive bacteria with a single DAC domain protein were analysed using this assay and all rapidly increased their c-di-AMP pools under these low osmolarity conditions which would trigger high turgor pressure (Fig 5C–5F).

We next examined the effect of an osmotic increase on c-di-AMP levels in several bacteria. These conditions would trigger lower turgor pressure. Ten minutes after glucose addition, either water, NaCl or KCl (0.1 M or 0.3 M final concentration) was added and cells were harvested after a further 10 minutes. The addition of NaCl or KCl resulted in rapid depletion or a block in c-di-AMP synthesis relative to water treated cells for all bacteria (Fig 5C–5F). We also tested the effect of non-ionic solutes (sorbitol and sucrose) on c-di-AMP levels in *L*. *monocytogenes* and found that they also stopped c-di-AMP synthesis or at higher levels triggered rapid c-di-AMP degradation (Fig 5G). These results demonstrate that environmental osmolarity changes trigger rapid c-di-AMP level fluctuation.

To determine the roles of CdaA and GdpP in c-di-AMP control in the energised cell assay, we examined an osmoresistant suppressor mutant of *Lc*. *lactis* Δ*gdpP* which has a partially defective *cdaA* (T273fs mutation). The c-di-AMP level did not vary over the 20 minute time course (Fig 5H). This demonstrates that CdaA and GdpP are the main controllers of c-di-AMP pool modulation in this assay.

## Discussion

The primary role of a second messenger is to transduce a signal(s) from the environment to effectors within the cell, resulting in a physiological response and ultimately adaptation. It is becoming apparent that a major role of c-di-AMP is in osmoregulation due to its control of osmolyte (K^+^ and compatible solute) transporters and observed osmosensitive and osmoresistant phenotypes of high and low c-di-AMP mutants, respectively. The results presented here reinforce this notion, with suppressor mutations which elevate K^+^ or glycine betaine transport rescuing osmoresistance in a high c-di-AMP mutant of *Lc*. *lactis*. Conversely in suppressor screens with mutants devoid of c-di-AMP, mutations inactivating osmolyte (peptides and glycine betaine) uptake [12, 39, 40] or increasing osmolyte (K^+^) export [9] have been found. Osmolyte import and export affects cellular turgor pressure and a recent proposition is that the central role of c-di-AMP is in the regulation of turgor pressure [41], which is supported by our work presented here.

The largest number of binding effectors under the control of c-di-AMP known at present are involved in K^+^ uptake [6, 7, 9]. KupB identified in this work has not been linked to c-di-AMP signalling pathways. It is a member of the Kup/HAK/KT family of K^+^ importer proteins (Pfam02705) which are widely distributed in bacteria, fungi and plants [28]. In bacteria, Kup homologs are most common in Proteobacteria (634 species), followed by Actinobacteria (91 species) in the Pfam database. Sixty-one species, mainly lactic acid bacteria, within the Firmicutes also contain Kup homologs. In *E*. *coli*, Kup is the major K^+^ importer under hyperosmotic conditions at low pH and likely functions as an H^+^—K^+^ symporter [42, 43]. Little is known regarding the regulation of Kup family proteins. Deletion of the intracellular the C-terminal domain of Kup in *E*. *coli* significantly reduced K^+^ transport activity [29] which suggests that this domain has a regulatory function. In Firmicutes, K^+^ transporters KtrAB, KtrCD, KdpABCD and KimA are regulated by c-di-AMP either via direct binding to the transporter or a two-component sensor kinase and/or through gene expression changes. In *Lc*. *lactis* MG1363, homologs of Ktr, Kdp and KimA are absent, and we did not find any evidence for c-di-AMP-mediated regulation of KupB. In lactoccoci, the *kup* gene has likely undergone a duplication event since immediately upstream there is a highly similar gene encoding KupA (>70% amino acid identity to KupB) in the majority of *Lc*. *lactis* strains. However, in strain MG1363 this is a pseudogene carrying a stop codon at amino acid 254. It was found that deletion of *kupB* did not affect the growth of *Lc*. *lactis* MG1363 in chemically defined media with lower K^+^ concentrations (S7 Fig), so it appears that there is another K^+^ transport system distinct from other characterised bacterial K^+^ transporters yet to be identified.

C-di-AMP is a significant regulator of compatible solute uptake through binding of the cystathionine-β-synthase (CBS) domain in the OpuCA carnitine transporters in *L*. *monocytogenes* and *S*. *aureus* [5, 8]. In our screen we identified a deletion event in *busR* which encodes the repressor of the glycine betaine transporter BusAA-AB [27]. It was found in previous work that *busAA-AB* expression was reduced in Δ*gdpP* [10], however the mechanism was not clear at that time. In this work, BusR was found to bind c-di-AMP and rescue osmoresistance in Δ*gdpP*, which has also been recently reported for *Streptococcus agalactiae* BusR [39]. It is likely that this is a conserved function in other Gram-positive bacteria that contain BusR orthologs with the same GntR family domain structure. C-di-AMP binding takes place via the TrkA_C domain with a K_d_ of 10 μM, which is higher than other c-di-AMP binding proteins analysed using DRaCALA. These include *L*. *monocytogenes* pyruvate carboxylase (8 μM), CbpA (2.2 μM), CbpB (1.8 μM), PstA (1.3 μM), OpuCA (1.2 μM) and PgpH (0.3–0.4 μM); *S*. *aureus* OpuCA (2.5 μM) and KdpD (2 μM); and *Enterococcus faecalis* OpuCA (6 μM) [5, 8, 16, 44, 45]. The homologous RCK_C domain of KtrA in *S*. *aureus* has a K_d_ of 0.4 μM [6]. Therefore it is possible that the affinities for proteins towards c-di-AMP have evolved to transduce the signal at different threshold concentrations of this nucleotide. We found that glycine betaine levels were significantly lower in the high c-di-AMP Δ*gdpP* mutant, which were restored by inactivation of *busR* or suppressor mutations which lower the c-di-AMP level. Low glycine betaine levels due to enhanced repression of *busAA-AB* by c-di-AMP bound BusR is a likely important contributor to osmosensitive phenotype of Δ*gdpP*. BusR promoter binding has also been found to be influenced by ionic strength *in vitro* [46], so it is possible that it senses multiple signals within the cell in order to respond to osmotic challenges.

Whilst the *kupB* and *busR* mutations resulted in restoration of osmolyte uptake to allow growth of Δ*gdpP* under high osmolarity, another way to achieve this is for the cell to simply reduce its intracellular c-di-AMP level. During this screen many destructive mutations in *cdaA* and restorative mutations in *gdpP* were observed which lowered the c-di-AMP level. Also in previous work a mutation in *glmM* was found to downregulate CdaA activity and lower the c-di-AMP level [11]. Two independent intergenic deletion mutations identified in the current work provide a straightforward way to lower the intracellular c-di-AMP pool, which is export. By removing a transcription terminator from a highly expressed ribosomal operon upstream, the suppressor mutants evolved to have a large increase in MDR gene expression. We were unable to demonstrate the MDR Llmg1210 alone was responsible for c-di-AMP export, since Llmg1211 co-expression was needed to stabilise the construct. Llmg1211 (DUF4811) has no homology to proteins with known function, but contains 2 N-terminal transmembrane domains and is located adjacent to Llmg1210 MDR homologs in other species suggesting they have a functional linkage. Interestingly *L*. *lactis* MG1363 contains another gene encoding a DUF4811 protein (Llmg1625), which is also located in an operon with MDR and MarR regulator genes upstream. C-di-AMP export has been observed in several pathogens where it triggers an IFN-β innate immune response [33, 47, 48]. In *L*. *monocytogenes*, overexpression of MDRs were observed following inactivation of their cognate transcriptional repressors, which led to elevated c-di-AMP export [32, 33]. A strain of *L*. *monocytogenes* (LO28) with a naturally occurring mutant *tetR* exported high levels of c-di-AMP because of strong expression of MdrT [34]. The amino acid identity (45%) between Llmg1210 and MdrT supports its likely function as a c-di-AMP exporter in *Lc*. *lactis*, however MdrT has also been shown to act as a bile exporter in *L*. *monocytogenes* [49] suggesting it may exhibit broad substrate specificity. Our results demonstrate that active c-di-AMP export is a mechanism by which the cell can modulate intracellular c-di-AMP levels significantly enough to impact cellular physiology, in this case osmoresistance.

Several other mutations were identified in the osmoresistance suppressor screen which triggered a lowering of the c-di-AMP level in Δ*gdpP*. The most common changes were in a peptide cleavage and export system which is broadly conserved in Gram-positive bacteria. Interestingly, loss of function mutations in Eep (RseP) and PptAB (EcsAB) genes were found during a screen for acid resistant suppressor mutants from a *ybbR* deletion strain of *S*. *aureus* [22]. Like that seen in our study, these mutations lowered the c-di-AMP compared to the parent strain. The connection between peptide processing/export and c-di-AMP level regulation is not clear at present. In *B*. *subtilis*, inactivation of EcsAB results in a defect in intramembrane cleavage activity by the Eep ortholog RsaP and it was suggested that peptides not cleared from the membrane may inhibit RsaP activity [50]. Peptides exported by these systems are known to function in cell-to-cell communication as pheromones following re-importation into a neighbouring cell [25, 51]. We tested the ability of spent supernatants from wild-type and Δ*gdpP Lc*. *lactis* to induce salt sensitivity in the *eep* or *pptAB* suppressor strains, but no effect was observed. Recent work in *L*. *monocytogenes* revealed that mutations in the Opp peptide uptake system can restore growth in a DacA (CdaA) mutant [12]. Peptides can function as osmolytes and were shown to be toxic in cells lacking c-di-AMP likely due to an uncontrollable increase in intracellular osmotic pressure [12]. Obtaining osmoresistant suppressor mutants unable to export peptides in Δ*gdpP* aligns with the hypothesis that elevated osmolyte (peptide) levels within a cell with high c-di-AMP can restore normal turgor pressure and allow growth on high salt agar. One additional mutation was also observed in each of two *kupB* suppressor mutants. These were in *ftsX* and *pptA* and at least for the latter, it is most likely this change which caused a lowering of the c-di-AMP level, similar to other single *ppt* mutants studied here. It remains to be determined if and how changes in FtsX, which regulates cell wall peptidoglycan hydrolase activity [52] affects c-di-AMP levels.

This study has identified both cellular and external stimuli which trigger significant variations in the c-di-AMP level in bacteria. It was found that enhanced K^+^ uptake due to gain-of-function mutations in the KupB transporter or simply overexpression of wild-type KupB resulted in elevated c-di-AMP in *Lc*. *lactis* and *Lb*. *reuteri*. This result is in agreement with recent work showing inactivation of the Trk K^+^ transporter gating component CabP which is predicted to lower K^+^ uptake, triggered a lower c-di-AMP level in *S*. *pneumoniae* [24]. Our results show that CdaA is activated as a result of higher K^+^ uptake during growth (since *gdpP* is inactivated in Δ*gdpP*). We also found that inactivation of BusR results in higher c-di-AMP suggesting that elevated glycine betaine uptake triggers c-di-AMP accumulation. In the cell suspension assay, under low osmotic conditions, c-di-AMP accumulation was observed under low osmotic conditions, but the addition of ionic or non-ionic solutes triggered a halt in synthesis or increased degradation of c-di-AMP. It therefore appears that the c-di-AMP level is modulated in response to turgor pressure changes as a result of water migration. Uncontrolled K^+^ or glycine betaine uptake during growth or low osmolarity conditions will result in water entering the cells causing high turgor pressure. In these cases, c-di-AMP accumulation would occur, allowing the cell to subsequently block uptake of K^+^ and compatible solutes. This then limits excessive cellular hydration and potentially cell lysis. Upon entry into a high osmolarity environment, cells require greater K^+^ and compatible solutes in order to prevent cellular dehydration and a fall in turgor pressure, so they therefore lower their c-di-AMP level to achieve this. This feedback loop ensures that the cell can quickly sense turgor pressure, and if need be, change the cell’s physiology to regulate water migration through the c-di-AMP signalling receptor network. These environmental changes are directly received at the protein/enzyme level, since the assay involves non-growing cells. Thus, the enzymes involved in c-di-AMP synthesis and degradation could therefore be considered as osmosensors in addition to their roles as osmoregulators.

The mechanisms underlying coordinated synthesis and/or hydrolysis of c-di-AMP in response to increased K^+^ and glycine betaine uptake, or external osmolarity, are currently unknown. Several possibilities appear possible. Both CdaA and GdpP contain 3 and 2 transmembrane domains which may allow sensing of membrane stretching or curvature which is likely to change under varying turgor pressures. CdaA forms a complex with and is regulated by the membrane bound extracellullar protein CdaR and the peptidoglycan biosynthesis enzyme GlmM in several bacteria [11, 19, 21, 23]. These protein-protein interactions (or individual activities) may be affected by changes in turgor pressure or ionic strength within the cell and affect c-di-AMP synthesis. In *L*. *lactis*, *cdaR* is a non-functional pseudogene, however c-di-AMP levels in this strain are responsive to external osmolarity changes, suggesting that this protein is not essential for sensing. Ultimately, identification of the osmo-signal sensing mechanism of the c-di-AMP system will be of significant interest as it is likely to be conserved across many bacteria.

## Materials and methods

### Bacterial culture conditions and suppressor screening

*Lc*. *lactis* strains (S1 Table) were grown at 30°C in M17 media (Difco, USA) supplemented with 0.5% (w/v) glucose (GM17). *Lb*. *reuteri* BR11 and *Lb*. *plantarum* 299v were grown in deMan Rogosa Sharpe (MRS) media (Oxoid, UK) at 37°C either anaerobically on agar or in static liquid cultures. *L*. *monocytogenes* ATCC 19112 and *S*. *aureus* IPOOM14235 were grown in Heart Infusion (HI) media (Oxoid, UK) at 37°C with aeration at 150 rpm. *E*. *coli* NEB-5α containing pRV300 derivatives were grown in Luria-Bertani (LB) broth containing 100 μg/ml ampicillin at 37°C with aeration at 230 rpm. *E*. *coli* NEB-5α containing pGh9 derivatives were grown in HI media (Oxoid, UK) containing 150 μg/ml erythromycin at 30°C with aeration at 230 rpm. Osmoresistant suppressor mutants of Δ*gdpP* strain OS2 were isolated and confirmed as described before [11]. Sanger sequencing of *cdaA* and *gdpP* from the suppressors and whole genome sequencing of 20 mutants using the HiSeq2000 platform was carried out at Macrogen (Seoul, South Korea). SNP analysis was carried out using Geneious 8.1.8. (Biomatters Ltd, New Zealand) as described previously [11].

### Genetic manipulation of strains

Plasmids and primers used in this study are shown in S2 and S3 Tables. *Lc*. *lactis* was transformed as described previously [10]. Insertional inactivated mutants were made using pRV300 and gene overexpression was done using pGh9. *Lc*. *lactis* transformants were grown at 30°C in the presence of 3 μg/ml erythromycin. *Lb*. *reuteri* was transformed as described previously [37] and plasmid containing cells were maintained using 10μg/ml erythromycin. For pGh9-*kupB*^*A618V*^ transformations into *Lc*. *lactis* 0.1–0.2M NaCl was added to the agar as a precaution to prevent mutations occurring. Wild-type or 42 amino acid deleted *busR* and downstream *busAA* promoter were cloned into pTCV-lac and introduced into *E*. *coli* T7 Express LysY (New England Biolabs) with selection using kanamycin (50 μg/ml). For β-galactosidase activity assays, strains were grown overnight in LB broth without NaCl (10 g/L tryptone; 5 g/L Yeast extract) and then diluted 1:100 in the same fresh LB medium and grown at 30°C, aeration 220 rpm to early log phase (OD_600_ ~0.25) where 0, 0.1, 0.2, 0.3 or 0.4 M NaCl was added. Following further incubation to OD_600_ ~0.6, cells were quantified for β-galactosidase as described previously (Miller, 1972), except chloroform and 0.1% SDS were used to permeabilize cells instead of toluene. The *busAA* promoter (252 bp) was also cloned into pTCV-lac and introduced into different *Lc*. *lactis* strains. Promoter activity (β-galactosidase activity) in different strains were compared following growth on GM17 0.1 M NaCl agar supplemented with 3 μg/ml erythromycin and 80 μg X-gal at 30°C for 2 days followed by storage at 4°C for 2 days for colour development. It should be noted that we have found that the Δ*gdpP* strain can undergo mutations restoring normal c-di-AMP levels during prolonged subculture even under normal growth conditions and caution should be taken when working with high c-di-AMP strains [53]. The Δ*gdpP* strains were generated with minimal sub-culturing and the *cdaA* and *gdpP* genes were checked for mutations and c-di-AMP levels were checked following mutant construction.

### Extraction and quantification of c-di-AMP

Strains were grown to mid-log phase (OD_600_ ~0.7), then pelleted by centrifugation (5,000 x g for 10 mins), cells then washed 2 times in 1/10 KPM buffer, then re-suspended in 1.5 ml ice-cold extraction buffer (40:40:20 methanol:acetonitrile:ddH_2_O v/v mix). Lysis was carried out according to that described previously [11]. For determining both extracellular and intracellular c-di-AMP levels, cells were grown in minimal media. First, overnight *Lc*. *lactis* cultures were diluted 1:100 into 15ml GM17 broth and incubated at 30°C till OD_600_ ∼ 0.7. Cells were pelleted by centrifugation at 5000 × *g* (Beckman Coulter, USA) for 10 min at 4°C, then washed 2 times and re-suspended in 15ml minimal media D6046 (Sigma-Aldrich, St. Louis, MO) supplemented with KH_2_PO_4_ 3.6mg/ml, K_2_HPO_4_ 7.3mg/ml_,_ histidine 0.13mg/ml, arginine 0.72 mg/ml, leucine 1mg/ml, valine 0.6mg/ml, glucose 0.5%, potassium acetate 0.9mg/ml, MOPS 13mg/ml, guanine 0.05mg/ml, xanthine 0.05mg/ml, FeSO_4_ 0.10mg/ml, ZnSO_4_ 0.1mg/ml_,_ adenine 0.2 mg/ml and incubated a further 3 hours. Cultures were centrifuged to separate supernatant and cells and supernatants were subsequently filtered (0.22 μm pore size) and used directly to quantify extracellular c-di-AMP. Cells were resuspended in 0.5 ml ice-cold extraction buffer and lysed as described previously [11]. C-di-AMP quantification using ultra performance liquid chromatography-coupled tandem mass spectrometry (UPLC-MS/MS) was carried out as described previously (11) using a different column (HSS PFP 1.8μm, 2.1 x 100 mm) and a BEH C18 VanGuard pre-column protector. Eluent A was composed of 0.1% of formic acid in water while eluent B was 100% acetonitrile. C-di-AMP was detected using electrospray ionization in a negative ion mode at m/z 657.5 → 328.26 and the internal standard 625.52 → 312.26 with collision energy being 30V and 28V, respectively.

### Quantification of intracellular K^+^ content

Overnight cultures were diluted 1:100 into GM17 broth with erythromycin if required and incubated until late log phase (OD_600_ ~ 1). Cultures (50 ml) were centrifuged at 5,000 x *g* (Beckman Coulter, USA) for 10 mins at 4°C and the supernatants were discarded. A second centrifugation was carried out and all media residue was removed by pipette. Cells were subsequently digested with 500μl of 15% HNO_3_ at 95°C for 1 hour. After digestion, the mixture was centrifuged at 5000 x *g* for 30 minutes at 4°C. Thereafter, the supernatant was collected to measure K^+^ content using Vista-Pro, CCD Simultaneous ICP-OES (Varian Inc., USA). The argon gas was ionized and used to create plasma at 7,000–10,000°C and the emission wavelength of 766.491 nm was used for measuring K^+^. Mean ± SEM were calculated based on three biological replicates.

### BusR expression, purification and DRaCALA

Full length BusR and the BusR C-terminal RCK_C domain were codon optimised for expression in *E*. *coli* and cloned into pRSETA as fusions to His-6 tags (Geneart, Germany). The C-terminal intracellular fragment of KupB was also cloned into pRSETA and pMAL-p5X. The resulting constructs were transformed into *E*. *coli* BL21 for expression. Briefly, bacterial cultures were grown at 37°C in LB broth with ampicillin (100 μg/mL) to OD ~ 0.7, then induced with 0.5 mM IPTG at 37°C for 3 hours. Bacteria were pelleted, resuspended in lysis buffer (30mM K_2_HPO_4_ pH 8, 300mM NaCl, 1mM PMSF), and lysed by sonication. For purification of the BusR C-terminal domain, after centrifugation the cell lysate was collected and applied to a Ni-NTA resin (Thermo Fisher), washed several times with wash buffer (30mM K_2_HPO_4_ pH 8, 300mM NaCl), and eluted with elution buffer (wash buffer + 300mM imidazole). The elute from the Ni-NTA resin was exchanged into nucleotide binding buffer (50mM Tris HCl pH 7.5, 150mM NaCl, 20mM MgCl_2_) using a PD10 desalting column (GE Healthcare). Radio-labeled c-di-AMP was prepared from ^32^P-ATP (Perkin-Elmer), and DRaCALA was performed as previously described [16]. Briefly, proteins were incubated with ^32^P-c-di-AMP for 10 minutes at room temperature, then spotted on a nitrocellulose membrane. Radioactivity was visualized with a Phospho-Imager and a Typhoon imaging system (GE Healthcare).

### Extraction and quantification of glycine betaine

*Lc*. *lactis* were grown overnight in GM17 at 30°C, subcultured 1:100 into 30 ml fresh GM17 and incubated at 30°C till OD_600_ ~ 0.9 (mid-log phase). Cells were harvested by centrifugation at 5200 x *g* for 10 min, and washed twice with 2ml 1/10 KPM buffer [0.01M K_2_HPO_4_ adjusted to pH 6.5 with H_3_PO_4_ and 1mM MgSO_4_.7H_2_O]. Cells were resuspended in 0.3 ml 1/10 KPM buffer and 1.2 ml extraction buffer (40% methanol:40% acetonitrile:20% ddH_2_O v/v). Samples were mixed with 0.5 ml equivalent of 0.1 mm zirconia/silica beads and disrupted using a Precellys 24 homogenizer (Bertin Technologies) three times for 30s each, with 1 min cooling on ice in between. Glass beads were separated by centrifugation at 17000 x g for 5 min. The supernatant was dried under liquid nitrogen before resuspended in 0.5 ml MilliQ water before filtered (0.22 mm pore size). Glycine betaine level was measured using UPLC/MS/MS with HSS PFP column (1.8μm, 2.1 x 100 mm; Waters) and a BEH C18 VanGuard pre-column protector. The method uses was the same with c-di-AMP quantification method described above, with some modifications. Glycine betaine was detected using electrospray ionization in positive ion mode at m/z 118 → 58 with the collision energy being 25eV. The levels were calculated based on a standard curve prepared with glycine betaine from Sigma-Aldrich.

### RNA extraction and RT-qPCR

Overnight cultures were diluted 1:100 in GM17 and incubated until OD_600_ ~ 0.6. To 500μL of culture, 1ml RNA protect reagent (Qiagen, Hilden, Germany) was added and then tubes were vortexed for 5s and held for 5 minutes at room temperature. Cells were harvested by centrifugation (5000 x *g* for 10min). RNA was extracted using the RNeasy minikit (Qiagen, Hilden, Germany) with some modifications as previously described [10]. cDNA was synthesized using SuperScript III First-Strand Synthesis SuperMix (Invitrogen, Carlsbad, CA). Platinum SYBR green quantitative PCR (qPCR) SuperMix-UDG (Invitrogen, Carlsbad, CA) was used for qPCR using the Rotor-gene Q qPCR machine (Qiagen) with primers described in S3 Table. Test genes *rplJ*, *llmg1209*, *llmg1210*, *llmg1211*, *llmg1212*, *cdaA* and *kupB* and the reference gene *tufA* were amplified along with no reverse transcriptase and no template controls. Data was analyzed using the comparative C_T_ method [54] from 3 biological replicates.

### Energised cell suspension assay method

Overnight cultures were diluted 1:100 in fresh media and incubated until OD_600_~0.7 (mid-log phase). Then, 30 ml of the mid-log culture was aliquoted into different tubes for various conditions (30 ml of culture was used for a single c-di-AMP measurement for each condition or time-point replicate). Next, cells were pelleted by centrifugation at 5,000 x *g* (Beckman Coulter, USA) for 10 min at 4°C and washed twice with buffer (1/10 KPM [0.01M K_2_HPO_4_ adjusted to pH 6.5 with H_3_PO_4_ and 1mM MgSO_4_.7H_2_O]). Cells were resuspended in 1.5 ml buffer and the cells were energised by adding 20mM D-glucose and incubated at 30°C (*Lc*. *lactis*) or 37°C (*Lb*. *plantarum*, *L*. *monocytogenes* and *S*. *aureus*) for 10 min to allow ATP production [37, 38]. D-deoxyglucose (20mM) was also tested as it de-energises cells and blocks ATP synthesis. Samples were either taken at this point for the time-course experiments or additional 90 μl ddH_2_O or NaCl (0.1M or 0.3M final concentration) or sorbitol (0.2M or 0.6M) or sucrose (0.2M or 0.6M) was added. At the 20 min time point the assay was stopped. To prevent any changes in external osmolarity or effects of centrifugation, at the harvest time point the cell suspensions were mixed directly with 0.5 ml equivalent of 0.1 mm zirconia/silica beads (Daintree Scientific, Australia) and were immediately lysed using a Precellys24 homogeniser (Bertin Instruments, France) three times for 30 seconds at 6,000 rpm with 1 min cooling on ice in between. Beads were separated by centrifugation at 16,873 x *g* (Eppendorf, Germany) for 5 min. The supernatant was mixed with methanol/acetonitrile producing a final v/v ratio of 40:40:20 methanol:acetonitrile:supernatant. The sample was centrifuged at 16,873 x *g* for 5 min and the supernatant was air-dried with nitrogen at 40°C before being resuspended in 0.5 ml ddH_2_O and filtered (0.22 μm pore size). Levels of c-di-AMP were determined with UPLC-MS/MS as above.

### Growth of WT *Lc*. *lactis* and Δ*kupB* in varying potassium concentrations

Overnight cultures of WT *Lc*. *lactis* and Δ*kupB* were diluted 1:100 in fresh GM17 media and grown OD_600_~0.7. Two millilitres of culture was centrifuged at 11000 x *g* for 3 mins and the cell pellets were washed 2 times in chemically defined media ZMB1 [55] with some modifications as follows. The potassium salts K_2_SO_4_ and KI were omitted and potassium phosphate buffers were replaced with NaH_2_PO_4_ (2.736 g/L) and Na_2_HPO_4_ (5.23 g/L). The washed cell pellets were re-suspended in 2 ml of modified ZMB1 and 5 μL of the cell suspension was inoculated into 5 ml of media with various concentrations of KCl (0.1 mM, 0.5 mM, 1 mM, 5 mM, 10 mM, 20 mM or 50 mM). The cultures were incubated at 30°C for 19 hours, at which point the OD_600_ was determined.

## Supporting information

S1 Fig (**A)** Location of suppressor mutations in Eep and PptAB (* = stop codon; fs = frameshift mutation). Secretion or lipoprotein signal sequences are cleaved by signal peptidase and the remaining signal peptides are digested by intramembrane protease Eep. The PptAB ABC exporter pumps the intramembrane peptides outside of the cell. **(B)** Comparison of growth of *Lc*. *lactis* MG1363 background strains on agar with varying NaCl following spotting of serial dilutions. The *pptB* gene was insertionally inactivated using pRV300 in Δ*gdpP*. **(C)** Levels of c-di-AMP (mean ± SEM) in *Lc*. *lactis* strains from three independent biological replications with significance shown (unpaired Student’s t test).
(DOCX)

S2 Fig DRaCALA of cell lysates containing the C-terminal intracellular domain of KupB fused to hexa-His or MBP tags with ^32^P-c-di-AMP. Negative and positive control cell lysates containing MBP or BusR, respectively, are included.
(DOCX)

S3 Fig (**A)** Expression of the *busAA* promoter in *E*. *coli* containing full length BusR or 42-amino acid deleted BusR under varying NaCl levels. **(B)** Domains present in different GntR transcriptional regulators including the BusR family containing the TrkA_C domain highlighted in pink.
(DOCX)

S4 Fig (**A)** Sequence showing the two deletion events (209-bp and 85-bp) occurring between *rplL* (highlighted yellow) and *rmaX* (highlighted green) in the osmoresistant suppressors. The larger deletion is coloured red (with purple in the middle), while the shorter deletion is coloured purple only. The two putative transcriptional terminators identified using the programs Erpin and RNAmotif through the online program ARNold are underlined and in bold. **(B)** Potential structure of the longer inverted repeat likely to be a transcriptional terminator. **(C)** Comparison of growth of strains (WT, Δ*gdpP* and the two osmoresistant suppressor mutants from Δ*gdpP*) on GM17 agar or GM17 agar + 0.2M NaCl following spotting of serial dilutions. **(D)** PCR confirmation of the two deletion events in the osmoresistant suppressor strains using primers flanking the deletions.
(DOCX)

S5 Fig Alignment of MDR protein Llmg1210 from *Lc*. *lactis* to known c-di-AMP exporting MDRs (MdrT and MdrM) from *L*. *monocytogenes* using CLUSTALW. Shaded residues (with asterisk below) are identical for all 3 proteins, while red text letters are identical across 2 proteins.
(DOCX)

S6 Fig (**A)** Gene arrangement in the Δ*gdpP rplL*^*termΔ85*^ osmoresistant suppressor mutant and pRV300 integrated variants. The putative transcription terminators downstream of *rplL* are missing in strain Δ*gdpP rplL*^*termΔ85*^ resulting in transcriptional read-through into the *rmaX* operon. Insertion of pRV300 sequentially through the *rmaX* operon blocks strong expression from the upstream *rplJ* promoter. **(B)** Levels of intracellular c-di-AMP (mean ± SEM) in *Lc*. *lactis* strains from three independent biological replications. *P* < 0.001 (***) and *P* < 0.01 (**) indicate significant differences compared to Δ*gdpP* (one-way ANOVA followed by Tukey’s test). NS = not significant. **(C)** Comparison of growth of pRV300 integrated strains on GM17 agar or GM17 agar + 0.2M NaCl following spotting of serial dilutions.
(DOCX)

S7 Fig Growth of WT *Lc*. *lactis* and Δ*kupB* in varying concentrations of K^+^. OD_600_ was measured following incubation for 19 hrs.
(DOCX)

S1 Table Bacterial strains used in this study. (DOCX)

S2 Table Plasmids used in this study. (DOCX)

S3 Table Primers used in this study. (DOCX)

S1 References References cited in S1 and S2 Tables. (DOCX)
