# Supplementary material for: Enhanced uptake of potassium or glycine betaine or export of cyclic-di-AMP restores osmoresistance in a high cyclic-di-AMP Lactococcus lactis mutant
Source: PLoS Genet. 2018 Aug 3;14(8):e1007574. doi: 10.1371/journal.pgen.1007574 (PMC6108528; doi:10.1371/journal.pgen.1007574)
Supplement: S1 Fig — (A) Location of suppressor mutations in Eep and PptAB (* = stop codon; fs = frameshift mutation). Secretion or lipoprotein signal sequences are cleaved by signal peptidase and the remaining signal peptides are digested by intramembrane protease Eep. The PptAB ABC exporter pumps the intramembrane peptides outside of the cell. (B) Comparison of growth of Lc. lactis MG1363 background strains on agar with varying NaCl following spotting of serial dilutions. The pptB gene was insertionally inactivated using pRV300 in ΔgdpP. (C) Levels of c-di-AMP (mean ± SEM) in Lc. lactis strains from three independent biological replications with significance shown (unpaired Student’s t test). (DOCX) [file pgen.1007574.s001.docx]

**Fig. S1**

**A**

 **B C**
