# Supplementary figures and images for: Enhanced uptake of potassium or glycine betaine or export of cyclic-di-AMP restores osmoresistance in a high cyclic-di-AMP Lactococcus lactis mutant
Source: PLoS Genet. 2018 Aug 3;14(8):e1007574. doi: 10.1371/journal.pgen.1007574 (PMC6108528; doi:10.1371/journal.pgen.1007574)

**Fig. S3**

**A**


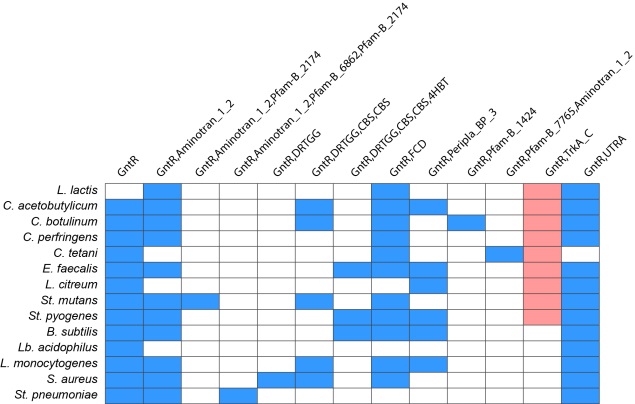


**B**

**Fig. S2**

Supplement: S3 Fig — (A) Expression of the busAA promoter in E. coli containing full length BusR or 42-amino acid deleted BusR under varying NaCl levels. (B) Domains present in different GntR transcriptional regulators including the BusR family containing the TrkA_C domain highlighted in pink. (DOCX) [file pgen.1007574.s003.docx]
