# Supplementary material for: Enhanced uptake of potassium or glycine betaine or export of cyclic-di-AMP restores osmoresistance in a high cyclic-di-AMP Lactococcus lactis mutant
Source: PLoS Genet. 2018 Aug 3;14(8):e1007574. doi: 10.1371/journal.pgen.1007574 (PMC6108528; doi:10.1371/journal.pgen.1007574)
Supplement: S4 Fig — (A) Sequence showing the two deletion events (209-bp and 85-bp) occurring between rplL (highlighted yellow) and rmaX (highlighted green) in the osmoresistant suppressors. The larger deletion is coloured red (with purple in the middle), while the shorter deletion is coloured purple only. The two putative transcriptional terminators identified using the programs Erpin and RNAmotif through the online program ARNold are underlined and in bold. (B) Potential structure of the longer inverted repeat likely to be a transcriptional terminator. (C) Comparison of growth of strains (WT, ΔgdpP and the two osmoresistant suppressor mutants from ΔgdpP) on GM17 agar or GM17 agar + 0.2M NaCl following spotting of serial dilutions. (D) PCR confirmation of the two deletion events in the osmoresistant suppressor strains using primers flanking the deletions. (DOCX) [file pgen.1007574.s004.docx]

**Fig. S4**

**A**

…***rplL***…AGCTAACGAAATCAAAGAAAAACTTGAAGCAGCTGGTGCTTCAATCACTCTTAAATAAGAATTGTTATCAATTTG**AAAAAACTGCCAAA**GTTA**TTTGGTGGTTTTTT**TGTTTTTTTTTAATAAAATAAAATTGATTCTTTAT**AAAATAACCCTCAGTATT**ATGA**AATACTGGGGGTTATTTT**TGTAAGATTTGCTTCTGAATTTATTCTATTCAGTTTGTGTAATATGTTGATGAAACGTTCTTAGAGTGGGAACCGTTATGATATTCCTAAAAAAACAATTGGTGTTATAAATAGTTTATGAATTACACACAAATAAAATATTAGTAAATCAAATAAAATAATTGACAATCTTCAAATAAAAGAATATCCTATAAATGTTAATATCGTTAGCAATGAAGCATAGTTAGCAAAAAAAGATTTTTTTCAGAAAGGAAACGTATGGATTATACAAAAGCAGCAGAAGACTTTTTCTCATGTATGAAGGCAAAAAAACGCAAAAAAAGTTTTGCTGAAACTCAATGAGAATGCTCAAGGTGAGCCTCTGGTTTTGGTTTATCTTTATA…***rmaX***


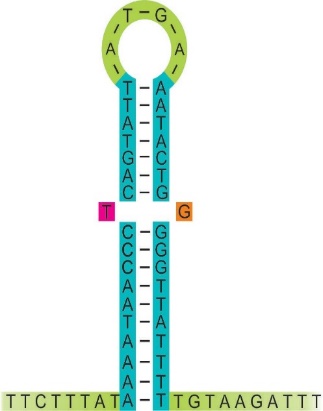


**D**

**B**

**C**
