# Supplementary material for: Enhanced uptake of potassium or glycine betaine or export of cyclic-di-AMP restores osmoresistance in a high cyclic-di-AMP Lactococcus lactis mutant
Source: PLoS Genet. 2018 Aug 3;14(8):e1007574. doi: 10.1371/journal.pgen.1007574 (PMC6108528; doi:10.1371/journal.pgen.1007574)
Supplement: S5 Fig — Shaded residues (with asterisk below) are identical for all 3 proteins, while red text letters are identical across 2 proteins. (DOCX) [file pgen.1007574.s005.docx]

**Fig. S5**
