# Supplementary material for: Enhanced uptake of potassium or glycine betaine or export of cyclic-di-AMP restores osmoresistance in a high cyclic-di-AMP Lactococcus lactis mutant
Source: PLoS Genet. 2018 Aug 3;14(8):e1007574. doi: 10.1371/journal.pgen.1007574 (PMC6108528; doi:10.1371/journal.pgen.1007574)
Supplement: S6 Fig — (A) Gene arrangement in the ΔgdpP rplLtermΔ85 osmoresistant suppressor mutant and pRV300 integrated variants. The putative transcription terminators downstream of rplL are missing in strain ΔgdpP rplLtermΔ85 resulting in transcriptional read-through into the rmaX operon. Insertion of pRV300 sequentially through the rmaX operon blocks strong expression from the upstream rplJ promoter. (B) Levels of intracellular c-di-AMP (mean ± SEM) in Lc. lactis strains from three independent biological replications. P < 0.001 (***) and P < 0.01 (**) indicate significant differences compared to ΔgdpP (one-way ANOVA followed by Tukey’s test). NS = not significant. (C) Comparison of growth of pRV300 integrated strains on GM17 agar or GM17 agar + 0.2M NaCl following spotting of serial dilutions. (DOCX) [file pgen.1007574.s006.docx]

**Fig. S6**

**A**

**C**

**B**
