# Supplementary material for: Enhanced uptake of potassium or glycine betaine or export of cyclic-di-AMP restores osmoresistance in a high cyclic-di-AMP Lactococcus lactis mutant
Source: PLoS Genet. 2018 Aug 3;14(8):e1007574. doi: 10.1371/journal.pgen.1007574 (PMC6108528; doi:10.1371/journal.pgen.1007574)
Supplement: S1 Table — (DOCX) [file pgen.1007574.s008.docx]

**Table S1**

**Bacterial strains used in this study**

| **Strains** | **Features** | **Antibiotic resistance** | **Source/Reference** |
| --- | --- | --- | --- |
| WT | *Lc. lactis* subsp. *cremoris* MG1363 |  |  |
| BR11 | *Lb. reuteri* BR11 |  | (Rush et al., 1994) |
| IPOOM14235 | *S. aureus* IPOOM14235 (methicillin resistant) |  | (Stephens et al., 2006) |
| 299v | *Lb. plantarum* 299v |  | Commercial probiotic product |
| ATCC 19112 | *L. monocytogenes* ATCC 19112 |  | ATCC |
| LysY | *E. coli* T7 Express LysY |  | New England Biolabs |
| Δ*gdpP* | This whole genome sequenced strain derived from WT has one spontaneous mutation (*gdpP^K122Stop^*) and a high c-di-AMP level. Also known as OS2. |  | (Zhu et al., 2016) |
| WT pGh9 | WT containing pGh9 | Em^r^ | This study |
| Δ*gdpP* pGh9 | Δ*gdpP* containing pGh9 | Em^r^ | This study |
| Δ*gdpP*Δ*pptB* | Δ*gdpP* containing inactivated *pptB* gene by integration of pRV300-Δ*pptB* | Em^r^ | This study |
| Δ*kupB* | WT containing inactivated *kupB* gene by integration of pRV300-Δ*kupB* | Em^r^ | This study |
| Δ*busR* | WT containing inactivated *busR* gene by integration of pRV300-Δ*busR2* | Em^r^ | This study |
| Δ*gdpP*Δ*busR* | Δ*gdpP* containing inactivated *busR* gene by integration of pRV300-Δ*busR1* | Em^r^ | This study |
| Δ*gdpP* pGh9-*kupB* | Δ*gdpP* overexpressing *kupB* from pGh9-*kupB* | Em^r^ | This study |
| Δ*gdpP* pGh9-*kupB^A618V^* | Δ*gdpP* overexpressing *kupB^A618V^* from pGh9-*kupB^A618V^* | Em^r^ | This study |
| BR11 pGh9-*kupB* | BR11 overexpressing *kupB* from pGh9-*kupB* | Em^r^ | This study |
| BR11 pGh9-*kupB^A618V^* | BR11 overexpressing *kupB^A618V^* from pGh9-*kupB^A618V^* | Em^r^ | This study |
| Δ*gdpP cdaA^T273fs^* | Suppressor mutant of Δ*gdpP* which contains a defective *cdaA* gene (frameshift in T273 codon) |  | This study |
| Δ*gdpP* pGh9-*rmaX* | Δ*gdpP* overexpressing *rmaX* using the *rplJ* promoter in pGh9-*rmaX* | Em^r^ | This study |
| Δ*gdpP* pGh9-*llmg1211* | Δ*gdpP* overexpressing *llmg1211* using the *rplJ* promoter in pGh9-*llmg1211* | Em^r^ | This study |
| Δ*gdpP* pGh9-*llmg1210-llmg1211* | Δ*gdpP* overexpressing *llmg1210* and *llmg1211* using the *rplJ* promoter in pGh9-*llmg1210-llmg1211* | Em^r^ | This study |
| Δ*gdpP rplL^termΔterm85^* | Suppressor mutant of Δ*gdpP* which contains a 85 nucleotide deletion in the terminator downstream of *rplL* | Em^r^ | This study |
| Δ*gdpP rplL^termΔterm85^* pRV300*-rplL* | Δ*gdpP rplL^termΔterm85^* with pRV300-*rplL* integrated blocking expression of downstream genes *rmaX*, *llmg1210* and *llmg1211* | Em^r^ | This study |
| Δ*gdpP rplL^termΔterm85^* pRV300*-rmaX* | Δ*gdpP rplL^termΔterm85^* with pRV300-*rmaX* integrated blocking expression of downstream genes *llmg1210* and *llmg1211* | Em^r^ | This study |
| Δ*gdpP rplL^termΔterm85^* pRV300*-llmg1210* | Δ*gdpP rplL^termΔterm85^* with pRV300-*llmg1210* integrated blocking expression of downstream gene *llmg1211* | Em^r^ | This study |
| Δ*gdpP rplL^termΔterm85^* pRV300*-llmg1211* | Δ*gdpP rplL^termΔterm85^* with pRV300-*llmg1211* integrated allowing expression of *rmaX*, *llmg1210* and *llmg1211* | Em^r^ | This study |
| LysY pTCV-lac-*busR-P_busAA_-lacZ* | *E. coli* containing pTCV-lac-*busR-P_busAA_-lacZ* to evaluate the ability of full length BusR to repress the *busAA* promoter. | Kan^r^, Em^r^ | This study |
| LysY-pTCV-lac *busR^Δ126^-P_busAA_-lacZ* | *E. coli* containing pTCV-lac *busR^Δ126^-P_busAA_-lacZ* to evaluate the ability of the 42-amino acid deletion variant of BusR to repress the *busAA* promoter. | Kan^r^, Em^r^ | This study |
