# Supplementary material for: Enhanced uptake of potassium or glycine betaine or export of cyclic-di-AMP restores osmoresistance in a high cyclic-di-AMP Lactococcus lactis mutant
Source: PLoS Genet. 2018 Aug 3;14(8):e1007574. doi: 10.1371/journal.pgen.1007574 (PMC6108528; doi:10.1371/journal.pgen.1007574)
Supplement: S2 Table — (DOCX) [file pgen.1007574.s009.docx]

**Table S2**

**Plasmids used in this study**

| **Plasmid** | **Properties** | **Antibiotic resistance** | **Source** |
| --- | --- | --- | --- |
| pGh9 | Previously called pPNG904. A pGhost9::IS*S1* derivative containing a Cm^r^ gene inserted into the EcoRI and SalI sites, replacing IS*S1*. Replicates at 30°C. | Em^r^, Cm^r^ | (Lo et al., 2009) |
| pGh9-*kupB* | pGh9 with entire *kupB* gene replacing the Cm^r^ gene | Em^r^ | This study |
| pGh9-*kupB^A618V^* | pGh9 with entire *kupB^A618V^* gene replacing Cm^r^ gene | Em^r^ | This study |
| pGh9-*rmaX* | pGh9 containing the *rplJ* promoter fused to *rmaX* for overexpression of *rmaX* | Em^r^ | This study |
| pGh9-*llmg1210* | pGh9 containing the *rplJ* promoter fused to *llmg1210* for overexpression of *llmg1210* | Em^r^ | This study |
| pGh9-*llmg 1211* | pGh9 containing the *rplJ* promoter fused to *llmg1211* for overexpression of *llmg1211* | Em^r^ | This study |
| pGh9-*llmg1210-llmg1211* | pGh9 containing the *rplJ* promoter fused to *llmg1210-llmg1211* for overexpression of *llmg1210-llmg1211* | Em^r^ | This study |
| pRV300 | Suicide vector in *Lc. lactis.* Replicates in *E. coli* | Ap^r^, Em^r^ | (Leloup et al., 1997) |
| pRV300-Δ*pptB* | pRV300 with 558-bp internal fragment of *pptB* for gene inactivation | Ap^r^, Em^r^ | This study |
| pRV300-Δ*kupB* | pRV300 with 792-bp internal fragment of *kupB* for gene inactivation | Ap^r^, Em^r^ | This study |
| pRV300-Δ*busR1* | pRV300 with 351-bp internal fragment of *busR* for gene inactivation | Ap^r^, Em^r^ | This study |
| pRV300-Δ*busR2* | pRV300 with 484-bp internal fragment of *busR* for gene inactivation | Ap^r^, Em^r^ | This study |
| pRV300-*rplL* | pRV300 with fragment containing *rplL* designed to integrate to prevent expression of *rmaX¸llmg1210* and *llmg1211* | Ap^r^, Em^r^ | This study |
| pRV300-*rmaX* | pRV300 with fragment containing *rmaX* designed to integrate to prevent expression of *llmg1210* and *llmg1211* | Ap^r^, Em^r^ | This study |
| pRV300-*llmg1210* | pRV300 with fragment containing *llmg1210* designed to integrate to prevent expression of  *llmg1211* | Ap^r^, Em^r^ | This study |
| pRV300-*llmg1211* | pRV300 with fragment containing *llmg1211* designed to integrate following *llmg1211*, not affecting expression of genes in this operon | Ap^r^, Em^r^ | This study |
| pTCV-lac | Shuttle vector with a promoterless lacZ gene. | Kan^r^, Em^r^ | (Poyart & Trieu-Cuot, 1997) |
| pTCV-lac-*busR-P_busAA_-lacZ* | pTCV-lac containg wild-type *busR* and the promoter of *busAA* fused to the *lacZ* reporter gene | Kan^r^, Em^r^ | This study |
| pTCV-lac *busR^Δ126^-P_busAA_-lacZ* | pTCV-lac containg 42 amino acid deleted version of *busR* and the promoter of *busAA* fused to the *lacZ* reporter gene | Kan^r^, Em^r^ | This study |
| pTCV-lac-*P_busAA_*_-_*lacZ* | pTCV-lac containg the promoter of *busAA* fused to the *lacZ* reporter gene | Kan^r^, Em^r^ | This study |
