# Supplementary material for: Enhanced uptake of potassium or glycine betaine or export of cyclic-di-AMP restores osmoresistance in a high cyclic-di-AMP Lactococcus lactis mutant
Source: PLoS Genet. 2018 Aug 3;14(8):e1007574. doi: 10.1371/journal.pgen.1007574 (PMC6108528; doi:10.1371/journal.pgen.1007574)
Supplement: S3 Table — (DOCX) [file pgen.1007574.s010.docx]

**Table S3**

**Primers used in this study**

| **Primer Name** | **Sequence (5ʹ to 3ʹ)** | **Target** |
| --- | --- | --- |
| 0448-F(B) | CCTGAACGTGAAGCTAGACG | Amplification and sequencing of *cdaA* |
| 0448-R | CGGTATATACTTCACCAGATTGG | Amplification and sequencing of *cdaA* |
| *gdpP*-F1 | TTGAGCATAACCAGTCGGCAC | Amplification and sequencing of *gdpP* |
| *gdpP*-F2 | CATCATCACGTCTATGGTGGTC | Sequencing of *gdpP* |
| *gdpP*-R | CAGGCAGGTAAATCAGCTGATGC | Amplification and sequencing of *gdpP* |
| *pptB*-F-KO | TAA**CTCGAG**ACGTCGTAACCTTTGGTACC | For cloning of a 558-bp internal fragment of *pptB* into pRV300 |
| *pptB*-R-KO | TAA**CTGCAG**CGATGACTGTGCGATTTGAG | For cloning of a 558-bp internal fragment of *pptB* into pRV300 |
| *pptB*-KO-int | GAGGTGACAGCCTTGGC | Primer upstream of *pptB* used to confirm integration |
| *kupB*-F-OE | ATT**CTCGAG**TTCACGAGCGAATTTCTAAGG | For cloning full length *kupB* or *kupB^A618V^* into pGh9 |
| *kupB*-R-OE | CCC**CTGCAG**GACAAGGCTGAATTCATCGCTC | For cloning full length *kupB* or *kupB^A618V^* into pGh9 |
| *kupB*-F-KO | AAA**CTCGAG**TAAATTGTTCGAGGACAGGGAGGAT | For cloning of a 792-bp internal fragment of *kupB* into pRV300 |
| *kupB*-R-KO | AAA**CTGCAG**TTAAATCGCGGCTAACGTTGACA | For cloning of a 792-bp internal fragment of *kupB* into pRV300 |
| *kupB-KO-int* | ATTGGATCC TTCACGAGCGAATTTCTAAGG | Primer upstream of *kupB* used to confirm integration |
| *kupB*_Cterm_XhoI_pRSETA | AAA**CTCGAG**TGGAATAAGTCACATAAGATTG | For cloning C-terminus of KupB into pRSETA plasmid |
| *kupB*_Cterm_EcoRI_pRSETA | AAA**GAATTC**TTAATTTGTTGCAGGAGCTTCTG | For cloning C-terminus of KupB into pRSETA plasmid |
| *kupB*-C-term-BamHI-pMAL | ATG**GGATCC**TGGAATAAGTCACATAAGATTG | For cloning C-terminus of KupB into pMAL-p5X plasmid |
| *kupB*-C-term-PstI-pMAL | AAA**CTGCAG**TTAATTTGTTGCAGGAGCTTCTG | For cloning C-terminus of KupB into pMAL-p5X plasmid |
| *busR-P_busAA_-*F | GTT**GAATTC**AGATGATAGCGGAAATGGAACG | For cloning *busR* and deleted *busR* and the *busAA* promoter into pTCV-lac |
| *P_busAA_-*F | CTT**GAATTC**CGTAGGAGCTTCTGATACAGG | For cloning the *busAA* promoter only into pTCV-lac |
| *busR-P_busAA_-*R | AAA**GGATCC**CATTCTATTACTCATGAGCCG | For cloning *busR* and deleted *busR* and the *busAA* promoter or the *busAA* promoter only into pTCV-lac |
| *busR*-F-KO | AAA**CTCGAG**GCAAGACAGCCAAGATACCAAC | For cloning of a 351-bp and 484-bp internal fragment of *busR* into pRV300 |
| *busR*-R-KO1 | AAA**CTGCAG**GATCAACCAATTTATCTAGG | For cloning of a 351-bp internal fragment of *busR* into pRV300 |
| *busR*-R-KO2 | AAA**CTGCAG**ATTGCAACAACTGTTGCTCCTG | For cloning of a 484-bp internal fragment of *busR* into pRV300 |
| *busR*-KO-int | GGGAGATGAGTAAACGTTATGG | Primer upstream of *busR* used to confirm integration |
| RUP | CAGGAAACAGCTATGAC | Plasmid specific primer used to confirm integration |
| *rplL*-int-F | AAA**CTGCAG**ACTGAAGCAGCATCTATCG | For cloning the *rplL* fragment into pRV300 |
| *rplL*-int-R | AAA**CTCGAG**CTCACCTTGAGCATTCTCA | For cloning the *rplL* fragment into pRV300 |
| *rmaX*-int-F | AAA**CTGCAG**ACTGAAGCAGCATCTATCG | For cloning the *rplL*-*rmaX* fragment into pRV300 |
| *rmaX-*int-R | AAA**CTCGAG**CCCATTAGTTCAGGCATTTA | For cloning the *rplL*-*rmaX* fragment into pRV300 |
| *llmg1210*-int-F | AAA**CTGCAG**ACATCAGCCTCTCCACAGC | For cloning the *llmg1210* fragment into pRV300 |
| *llmg1210*-int-R | AAA**CTCGAG**GTATCATGATTTTTGACCTCC | For cloning the *llmg1210* fragment into pRV300 |
| *Llmg1211*-int-F | AAA**CTGCAG**CACAGGAAATATCCTTGGC | For cloning the *llmg1211* and intergenic region upstream *llmg1212* fragment into pRV300 |
| *Llmg1211-*int-R | AAA**CTCGAG**GTGCTTGCTATAAAACAACTCC | For cloning the *llmg1211* and intergenic region upstream *llmg1212* fragment into pRV300 |
| *P_rplJ_-*F | GTT**CTGCAG**TCGGTCTTGCTTATACTAGAATC | For cloning the putative strong promoter from *rplJ* (*P_rplJ_*) |
| *P_rplJ_-rmaX-R* | GCTAACTATGCTTCATTGCT TTATGCAACAACGATAGATGCTGCTTC | For cloning the putative strong promoter from *rplJ* (*P_rplJ_*) fused with *rmaX* |
| *rmaX-*F | AGCAATGAAGCATAGTTAGC | For cloning *rmaX* into pGh9 as a fusion to the *P_rplJ_* |
| *rmaX-*R | AAA**CTCGAG**TCCAGCAAAGGTCCCAATCA | For cloning *rmaX* into pGh9 as a fusion to the *P_rplJ_* |
| *P_rplJ_-llmg1210-R* | GAGATGCGAGTAATGGTACG TTATGCAACAACGATAGATGCTGCTTC | For cloning the putative strong promoter from *rplJ* (*P_rplJ_*) fused with *llmg1210 and llmg1210-llmg1211* |
| *llmg1210-*F | CGTACCATTACTCGCATCTC | For cloning *llmg1210* into pGh9 as a fusion to the *P_rplJ_* |
| *llmg1210-*R | AAG**CTCGAG**CTGTGGAACCTTTGATGTAG | For cloning *llmg1210* into pGh9 as a fusion to the *P_rplJ_* |
| *P_rplJ_-llmg1211-R* | GTACAGGCGCTGGTGCGACC TTATGCAACAACGATAGATGCTGCTTC | For cloning the putative strong promoter from *rplJ* (*P_rplJ_*) fused with *llmg1211* |
| *llmg1211-*F | GGTCGCACCAGCGCCTGTAC | For cloning *llmg1211* into pGh9 as a fusion to the *P_rplJ_* |
| *llmg1211-*R | AAT**CTCGAG**GTGACCTTTATAGAGCAAACAG | For cloning *llmg1211* into pGh9 as a fusion to the *P_rplJ_* |
| *llmg1210-llmg1211-*F | CGTACCATTACTCGCATCTC | For cloning *llmg1210-llmg1211* into pGh9 as a fusion to the *P_rplJ_* |
| *llmg1210-llmg1211-*R | AAT**CTCGAG**GTGACCTTTATAGAGCAAACAG | For cloning *llmg1210-llmg1211* into pGh9 as a fusion to the *P_rplJ_* |
| *cdaA*-F | CCGTGACAAGTGCCTACCTT | For RT-qPCR of the *cdaA* gene |
| *cdaA*-R | GTGCTACTGAGATTCCGCCA | For RT-qPCR of the *cdaA* gene |
| *kupB*-F | TAGGACATGTAGGTCGCGGA | For RT-qPCR of the *kupB* gene |
| *kupB*-R | GTGTGCTAAAAGCCAAGCCC | For RT-qPCR of the *kupB* gene |
| *rplJ*-F | ACTTCGTGAAGCAGGCGTTG | For RT-qPCR of the *rplJ* gene |
| *rplJ*-R | GCTACTGCTGATGGGCCAGA | For RT-qPCR of the *rplJ* gene |
| *rmaX*-F | TGCTCAAGGTGAGCCTCTGG | For RT-qPCR of the *rmaX* gene |
| *rmaX*-R | AGAATATTTGCAACTCGTGCGGT | For RT-qPCR of the *rmaX* gene |
| *llmg1210*-F | AGTGATGGCGATGGGCGTTG | For RT-qPCR of the *llmg1210* gene |
| *llmg1210*-R | GTCCAATCACAAGTCCGCCCA | For RT-qPCR of the *llmg1210* gene |
| *llmg1211*-F | CCTTGGCGGAATTGCTACCCT | For RT-qPCR of the *llmg1211* gene |
| *llmg1211*-R | CAGCGGCAGAATAAATCGTCGT | For RT-qPCR of the *llmg1211* gene |
| *llmg1212-F* | AGCGGTCTTGAACCGATTGT | For RT-qPCR of the *llmg1212* gene |
| *llmg1212-F* | CAGCCTCAATATGCGCAAGC | For RT-qPCR of the *llmg1212* gene |
| *tufA-F* | GGTAGTTGTCGAAGAATGGAGTGTGA | For RT-qPCR of reference gene *tufA* |
| *tufA-R* | TAAACCAGGTTCAATCACTCCACACA | For RT-qPCR of reference gene *tufA* |
