# Supplementary material for: Enhanced uptake of potassium or glycine betaine or export of cyclic-di-AMP restores osmoresistance in a high cyclic-di-AMP Lactococcus lactis mutant
Source: PLoS Genet. 2018 Aug 3;14(8):e1007574. doi: 10.1371/journal.pgen.1007574 (PMC6108528; doi:10.1371/journal.pgen.1007574)
Supplement: S1 References — (DOCX) [file pgen.1007574.s011.docx]

**Supplementary references:**

Leloup, L., Ehrlich, S. D., Zagorec, M., & Morel-Deville, F. (1997). Single-crossover integration in the *Lactobacillus sake* chromosome and insertional inactivation of the *ptsI* and *lacL* genes. *Appl Environ Microbiol, 63*(6), 2117-2123.

Lo, R., Turner, M. S., Barry, D. G., Sreekumar, R., Walsh, T. P., & Giffard, P. M. (2009). Cystathionine gamma-lyase is a component of cystine-mediated oxidative defense in *Lactobacillus reuteri* BR11. *J Bacteriol, 191*(6), 1827-1837.

Poyart, C., & Trieu-Cuot, P. (1997). A broad-host-range mobilizable shuttle vector for the construction of transcriptional fusions to beta-galactosidase in Gram-positive bacteria. *Fems Microbiology Letters, 156*(2), 193-198.

Rush, C. M., Hafner, L. M., & Timms, P. (1994). Genetic modification of a vaginal strain of *Lactobacillus fermentum* and its maintenance within the reproductive tract after intravaginal administration. *J Med Microbiol, 41*(4), 272-278.

Stephens, A. J., Huygens, F., Inman-Bamber, J., Price, E. P., Nimmo, G. R., Schooneveldt, J., . . . Giffard, P. M. (2006). Methicillin-resistant *Staphylococcus aureus* genotyping using a small set of polymorphisms. *J Med Microbiol, 55*(Pt 1), 43-51.

Zhu, Y., Pham, T. H., Nhiep, T. H. N., Vu, N. M. T., Marcellin, E., Chakrabortti, A., . . . Turner, M. S. (2016). Cyclic-di-AMP synthesis by the diadenylate cyclase CdaA is modulated by the peptidoglycan biosynthesis enzyme GlmM in *Lactococcus lactis.* *Mol Micro, 99*(6), 1015–1027.
